# Transformation of traditional knowledge of medicinal plants: the case of Tyroleans (Austria) who migrated to Australia, Brazil and Peru

**DOI:** 10.1186/1746-4269-8-44

**Published:** 2012-11-16

**Authors:** Heidemarie Pirker, Ruth Haselmair, Elisabeth Kuhn, Christoph Schunko, Christian R Vogl

**Affiliations:** 1 Working Group: Knowledge Systems and Innovations, Division of Organic Farming, Department for Sustainable Agricultural Systems, University of Natural Resources and Life Sciences, Vienna, Austria

**Keywords:** Medicinal plants, Traditional knowledge, Migration, Quantitative ethnobotany, Informant consensus

## Abstract

**Background:**

In ethnobotanical research, the investigation into traditional knowledge of medicinal plants in the context of migration has been of increasing interest in recent decades since it is influenced and changed by new environmental and social conditions. It most likely undergoes transformation processes to match the different living circumstances in the new location. This study compares the traditional knowledge of medicinal plants held by Tyroleans – and their descendants – who emigrated to Australia, Brazil and Peru at different time scales. The study’s findings allow a discussion of the complexities and dynamics that influence this knowledge within the context of long-distance migration.

**Methods:**

Information was obtained from 65 informants by free-listing, semi-structured interviews and non-participatory observation in Tyrol (Austria) and the migrants’ countries: Australia, Brazil and Peru. The collected data was analysed using different quantitative approaches, including statistical tests, and compared between the countries of investigation.

**Results:**

All respondents in all four investigation areas claimed that they had knowledge and made use of medicinal plants to treat basic ailments in their day-to-day lives. Informants made 1,139 citations of medicinal plants in total in free lists, which correspond to 164 botanical taxa (genus or species level) in Tyrol, 87 in Australia, 84 in Brazil and 134 in Peru. Of all the botanical taxa listed, only five (1.1%) were listed in all four countries under investigation. Agreement among informants within free lists was highest in Tyrol (17%), followed by Peru (12.2%), Australia (11.9%) and Brazil (11.2%). The proportion of agreement differs significantly between informants in Australia and Tyrol (p = 0.001), Brazil and Tyrol (p = 0.001) and Peru and Tyrol (p = 0.001) and is similar between informants in the migrant countries, as indicated by statistical tests. We recorded 1,286 use citations according to 744 different uses (Tyrol: 552, Australia: 200, Brazil: 180, Peru: 357) belonging to 22 different categories of use. Use values are significantly different between Tyrol and Australia (p < 0.001) but not between Tyrol and Brazil (p = 0.127) and Tyrol and Peru (p = 0.853). The average informant agreement ratio (IAR) in Tyrol is significantly higher than in Australia (p = 0.089) and Brazil (p = 0.238), but not Peru (p = 0.019).

**Conclusions:**

Changing ecological and social conditions have transformed and shaped traditional knowledge of medicinal plants through adaptation processes to match the new circumstances in the country of arrival. Continuation, substitution and replacement are strategies that have taken place at different rates depending on local circumstances in the research areas. Traditional knowledge of medicinal plants acquired in the home country is continuously diminishing, with its composition influenced by urbanisation and ongoing globalisation processes and challenged by shifts from traditional healing practices to modern healthcare facilities.

## Background

The global increase in migration over the past few decades [1] has drawn the attention of ethnobotanists to the effects of these events on ethnobotanical knowledge and related traditional healing practices among migrant communities [2-4]. Over the past few years, numerous cross-cultural studies among migrants of ethnic groups have been conducted e.g. in the United States [5-8], in Cuba [3,9] and in Europe [10-21] to provide a better understanding of the dynamics of medicinal plant use by migrants engaged in international or national migration processes. In the course of these studies, it became obvious that the dynamic social interaction between migrants and host societies is strongly influenced by changing natural and social environments, e.g. changing health conditions and different healthcare systems to which the newcomers are exposed [2,22].

This study focuses on the traditional knowledge of medicinal plants of Tyroleans living in the Austrian province (*Bundesland*) of Tyrol, and Tyroleans and their descendants who emigrated to Australia (from the 1950s), Brazil (between 1933 and 1938) and Peru (in 1859 and 1868). Throughout the history of Austrian migration there have been significant regional differences in Austria. Although the migration patterns of the federal state of Tyrol are statistically similar to those of Burgenland, Vienna and Lower Austria, the Tyrolean emigration projects are well known [23]. The immigration countries for the research project were selected to represent i) different social milieus and structures of settlement, ii) different economic conditions, iii) different environmental conditions iv) different periods of immigration and v) sizeable Tyrolean emigrant populations. Traditional knowledge (TK) is a “cumulative body of knowledge, practice and belief, evolving by adaptive processes and handed down through generations by cultural transmission, about the relationship of living beings (including humans) with one another and with their environment” [24] in rural, semi-urban and urban communities. These adaptive processes tend to be experimental, dynamic and closely related to a way of life in a particular geographic area. This includes the processes whereby knowledge is generated, stored, applied and transmitted to others within specific social-ecological contexts [25]. The term “traditional” refers to cultural and historical continuity in a group’s resource use practice and includes the transmission of knowledge on health practices based on ethnobotanical knowledge [24]. The impact of migration events on cultural-related health practices are many and complex and may severely challenge beliefs, values, knowledge, technology, exchange systems and the use of natural resources since the application of traditional knowledge most often depends on continued access to specific land and resources [26,27]. Previous studies state that ethnobotanical knowledge changes as it is transferred and appropriated by people in order to adapt to new surroundings and changing environments [3,4,9,27-29]. The new context might contribute to the creation of new knowledge and practices in the area of arrival as migrants exchange knowledge, cultural traditions and medicinal plants with the local population [22,30]. Therefore, the level of the migrants’ knowledge can turn out to be even higher or more diverse than the knowledge they initially had before emigration. Migration is actually one of the main drivers by which plants and associated ethnobotanical knowledge are, and have been, dispersed across the globe [3]. Some of the traditional knowledge of medicinal plants taken with them might continue to be applied if there is access to the desired plant resource or the natural conditions for the designated plant are available [31]. Therefore, depending on the specific context and conditions of migration, the following two processes take place: (1) adaptation to the new flora of the host country by substituting and/or incorporating plants into health practices and (2) continued use of plants that grow in both host and home environments or acquisition of the desired plant from the migrants’ home countries through importation and cultivation [3,4].

This study aims to explore the transformations in migrant Tyroleans’ knowledge of medicinal plants that have occurred during their migration history. Ethnobotanical studies [32-35] undertaken in Tyrol in Austria have shown that people in Tyrol still have traditional knowledge about the medicinal use of plants. So far most studies undertaken on long-distance or international migration have examined the impacts of migration on medicinal plant knowledge of groups of people who have moved from the tropics to temperate countries [4]. The focus of this study is on a subject that has not received much attention until now: the long-distance migration of groups of people from a temperate area to temperate, subtropical and tropical areas of the world at different times.

The data collected from Tyroleans and Tyrolean migrants and their descendants in four different areas of the world will be presented and discussed regarding 1) the traditional knowledge of medicinal plants and their uses, 2) knowledge distribution and variation, and 3) the continuation and adaptation of traditional medicinal plant knowledge over time. Quantitative comparisons of the knowledge generated within the various field sites should provide a better understanding of the complexities and dynamics of medicinal plant knowledge within the context of long-distance migration. Based on the results obtained, the study’s findings will be explored regarding the cultural and environmental forces that have shaped knowledge of medicinal plants among migrants with a Tyrolean cultural background.

## Methods

### Ethnogeography and biogeography of research sites

#### Austria Tyrol

**Figure 1:**
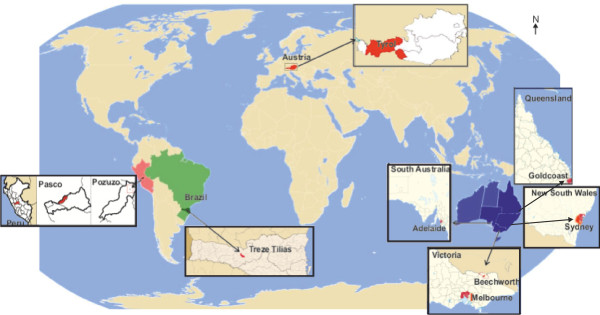
Research sites.

Tyrol is the third largest federal state in Austria covering an area of 12,640 km^2^ and a population of 704,472 [36] (Figure 1). It is situated in the Alps (465 m to 3,798 m above sea level) and is characterised by a temperate inner alpine climate with subcontinental influences. Valleys with narrow and remote side valleys shaped by high mountain ranges form its landscape. The main structure of settlement consists of villages, a few small cities (up to 20,000 inhabitants) and the region’s capital, Innsbruck, which has a population of 118,035 [36]. Besides small and medium-sized industries (textiles, glass, metal processing and food production), tourism provides the main source of income. The alpine landscape is predominantly characterised by spruce forests and alpine pastures. Farmers’ gardens in mountainous alpine areas are a typical element of land use within the mosaic of agroecosystems managed by farmers [37]. Since farming systems have undergone a process of change over the last few decades they are now seen as economically less important, but still play a major role in Tyrolean’s view of themselves and the maintenance of the countryside. Until the 1950s, the predominantly peasant community had to rely on medicinal plants as they lived in remote areas where there was a lack of medical care. Owing to limited means and poor transportation, doctors were only called out in serious cases and therefore people had to rely on their own or local experts’ knowledge of medicinal plants growing in their surroundings [33]. This knowledge has changed significantly due to changes in people’s socioeconomic situations and improvements in national healthcare facilities due to ongoing industrialisation and globalisation [35]. Austria has a compulsory state-funded healthcare system along with the option of private healthcare which provides a large, high-quality network of doctors and hospitals all over the country. Overall health in Austria is among the best in developed countries. Life expectancy at birth is 78 for men and 83 for women. The major causes of mortality are diseases of the circulatory system (50%) and neoplasms (23%) which are patterns of disease similar to those in other developed countries [38]. Medicinal plants for self-medication are now no longer essential, but offer people a popular alternative to conventional health practices such as the use of pharmaceuticals, healthcare professionals and medical facilities. Medicinal plants in Europe, their extracts, active components and finished products have been described in many national pharmacopoeias that have ultimately led to a unified European Pharmacopoeia (EP), setting the standards in Europe for the use of these products as drugs. The study sites in Tyrol were chosen to represent the areas from where most of the migrants who moved to Australia, Brazil and Peru came. Most of the migrants living in Treze Tílias today came from Wildschönau (47 27^′^0″N, 12°3^′^0″O), which is in the Upper Inn Valley area. [38]. Many of those living in Pozuzo today came from Silz (47°16^′^0″N, 10°55^′^60″E) in the Lower Inn Valley. Both sites are in Western Tyrol. We also carried out field research in Eastern Tyrol/Lienz (46 49^′^47″N, 12°46^′^11″E) from where some of the migrants to Australia came.

#### Australia New South Wales, Queensland, South Australia and Victoria

After the Second World War, Austria’s poor economic situation provided a strong incentive for emigration. However, many of those who wanted to emigrate did not have the financial means to do so [39]. The Assisted Passage Scheme (APS) was the only opportunity for some Tyrolean applicants to relocate to a new country [40]. The APS agreement was signed between the Australian Government and the Provisional Government in Austria in 1952 and allowed Austrians to apply for immigration. Immigrants could travel to Australia for a nominal sum of ten British pounds, provided they signed a contract to work for two years in a job allocated to them by the Australian Government [39]. Many Austrian migrants wanted to improve their living standards and between 1952 and 1961 almost 17,000 Austrians took advantage of this opportunity. Most of the migrants were in their early twenties when they read newspaper advertisements offering them a new start in Australia. A small number of unassisted immigrants who paid for the journey themselves also moved to Australia. It is not clear how many Tyroleans migrated since 1948 as no statistical records are kept for specific Austrian provinces (*Bundesländer*). The National Archives of Australia [41] holds 173 migration records of Austrians born in Tyrol who migrated to Australia between 1948 and 1967. After 1967 migration records were only ordered alphabetically, with the country of departure no longer indicated, making it impossible to determine the exact number of Tyroleans who migrated. From 1959 the pattern of Austrian migration became more individualistic and the number of migrants decreased. However, in 2008 the estimated resident population of Austrians born in Austria is 20,828 [42]. Before the 1970s most Austrians settled in New South Wales (36%), followed by Queensland (11.6%) and South Australia (10%). The interviewees live in the greater metropolitan area of Sydney (33°53^′^S, 51°12 O) in New South Wales, on the Gold Coast (27°59^′^S, 153°22^′^O) in Queensland, in Adelaide (34°55^′^0″S, 138°36^′^0″E) in South Australia and in the greater metropolitan area of Melbourne (37°50^′^S, 145°00^′^O) and in Beechworth (36 21^′^0″S, 146°41^′^0″E) in Victoria (Figure 1).

Austrian migrants in Australia have not formed separate communities and mainly live dispersed throughout the urban and peri-urban areas of Australia. Having learnt English, the Austrian migrants became assimilated fairly quickly and participated in creating the identity of what is now a multicultural Australian society, together with numerous migrants from other European countries in the 1950s. Like other ethnic groups who migrated to Australia, Austrian migrants set up Austrian national clubs in the main cities where Austrian culture is still celebrated today, predominantly by older members, through various club activities. Many of the respondents regularly travel to Austria and maintain contact with their Austrian relatives and friends through the modern convenience of internet applications (email and Skype, for example).

Depending on the research site, climatic conditions range from a temperate climate in the south and east (South Australia and Victoria) to subtropical (New South Wales) and tropical conditions in the north (Queensland). Many species of plants in Australia are found nowhere else on earth (more than 80% are endemic to the country), except where they have been introduced by humans. The high diversity of flora includes large numbers of species in ecologically significant genera such as Acacia, Eucalyptus, Melaleuca, Grevillea and Allocasuarina [43]. The Australian population generally has a good health record, with life expectancy one of the highest among developed countries (M: 79; F: 84) [44]. However there are some groups with a poor health status, notably Aboriginal people and Torres Strait Islanders who now only make up 2% of the population. Otherwise the pattern of disease is similar to that of other developed countries. Some of the common health concerns in Australia are skin cancer, heat strokes, obesity, diabetes, dengue fever and other chronic diseases common in developed countries [44]. Healthcare in Australia follows Western traditions with technical and scientific skills used to prevent, examine and treat illness. The Australian government provides help with medical expenses and hospital care through a scheme called Medicare which is Australia’s public healthcare system designed to give access to free or low cost medical, optometric and hospital care [44]. Besides the dominant healthcare system of biomedicine, western herbal medicine is the most widely used form of complementary healthcare. European herbal medicine was introduced to Australia by the first European settlers and has remained influenced by European rather than indigenous medical practices [45]. It is provided professionally by qualified herbalists and naturopaths and largely uses plants native to Europe. Indigenous herbal medicine, although still used to some extent by aboriginal Australians, has no broad usage in mainstream Australian society at present [45,46]. The herbal medicine market in Australia has experienced rapid growth in the past ten years with herbal products being available from supermarkets, pharmacies, health food stores and by mail order.

#### Brazil Treze Tílias

**Figure 2:**
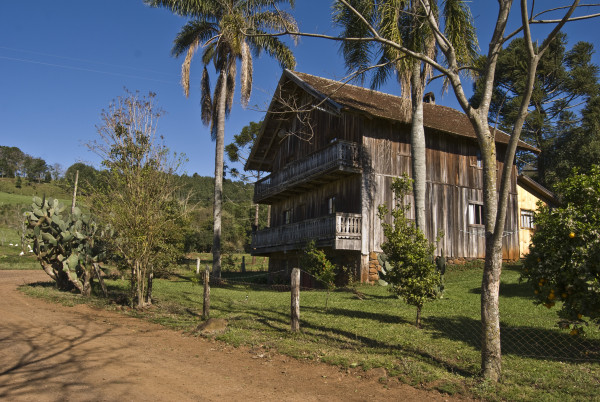
A Tyrolean-style farmer’s house in Treze Tílias (Photo: Elisabeth Kuhn).

Treze Tílias (27°0^′^0″S, 51°24^′^0″W) is a municipality in Santa Catarina covering an area of 185.205 km^2^ in Southern Brazil (796 m above sea level) (Figure 1) with a population of 6,341, of whom 4,715 in urban areas and 1,626 in rural areas [47]. The population now consists mainly of descendants of people of Italian, German, Austrian and Japanese origin with just 0.13% of the population comprising indigenous people of the region. 74% of the population live in the centre of the settlement, while the remainder live as farmers in the surrounding rural area. The landscape is characterised by hills and determined by temperate climatic conditions. The natural vegetation is composed of mixed Araucárian *(Araucaria angustifolia)* forests. Agricultural activities are important in the region, with corn as the dominant crop. Other agricultural goods are soya, grapes, mate tea, manioc, wheat, black bean, oranges and rice. Between September 1933 and January 1938, 789 Austrians – of whom 560 were Tyrolean –moved to Treze Tílias. Over 300 of the migrants came from the Lower Inn Valley in Tyrol [48,49]. The foundation of the “Tyrolean Brazil” goes back to a migration project initiated by the Austrian Minister of Agriculture, Andreas Thaler, and was funded by the Austrian Government. The project was designed to allow émigré Austrians to maintain their customs and traditions. A location was chosen that was said to be free of other populations and a long way from the next city in order to avoid “cultural assimilation” [50]. Farmers predominantly were allowed to take part in the project because they were thought to be used to hard work and able to live in harsh living conditions [51]. These preconditions were seen as the best qualifications for establishing an Austrian colony in Brazil. When migrants arrived in the Treze Tílias area, the infrastructure was barely developed and migrants had more or less to build the settlement themselves (Figure 2). In the early years, migrants practised subsistence farming and lived scattered throughout the municipality. The outbreak of the Second World War halted immigration from Austria but official contact with Austria was re-established after the end of the Second World War. Ethnic tourism was introduced, supported by the Tyrolean government, and led to an improvement in the economic situation [49,52,53]. A telenovela filmed in Treze Tílias and broadcast on public television across Brazil in 1991 made “Tyrolean Brazil” famous. Today, Treze Tílias is considered a tourist location predominantly for Brazilians. A range of restaurants offer typical Austrian-Tyrolean food, Tyrolean dances, music and other traditions for tourists.

In 2004 life expectancy in Santa Catarina was 73 (M: 69; F: 77) and the area had Brazil’s lowest infant mortality rate and was equipped with modern healthcare facilities (two hospitals) [47]. Since there was no hospital or pharmacy in Treze Tílias before the arrival of the Austrians, medical care was provided by four Austrian nuns who also migrated to Treze Tílias to take care of the migrants’ education and health needs [54]. Although modern medical facilities are now the predominant form of healthcare in Treze Tílias, medicinal plants are still recognised as being valuable for healing purposes. Local pharmacies therefore offer a wide range of medicinal plants in the form of herbal teas coming from all over Brazil. This attitude is reflected in the habit of many informants of consulting a medicinal plants expert who is a woman of Italian origin. Several medicinal plants such as *Matricaria chamomilla*, *Calendula officinalis*, *Rosmarinus officinalis*, *Ocimum. basilicum* and *Origanum vulgare* were introduced and adapted by immigrants of mainly Italian, German and Austrian origin [55,56]. Brandão’s surveys [55,57] reflect that Brazilian flora offers one of the world’s richest sources of medicinal plants due to its biodiversity. The accelerated expansion of pharmaceutical production in Brazil since the Second World War has led to medicinal plants and botanical products native to the country being replaced by synthetic products and foreign plants within the Brazil’s Official Pharmacopoeia. Today, Brazil remains an important supplier of botanical raw material for the international pharmaceutical market.

#### Peru Pozuzo

**Figure 3:**
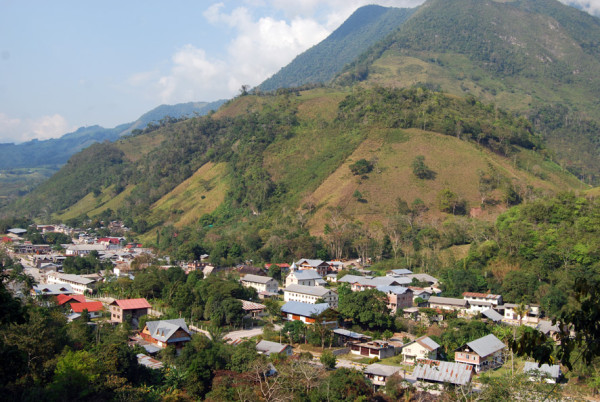
View of Pozuzo (Photo: Ruth Haselmair).

Pozuzo (10°4^′^0″S, 75°32^′^0″W) is a district in the Department of Oxapampa in the Central Andean region (750 m above sea level) of Pasco in Peru (Figure 1). Pozuzo has a tropical climate. 170 Tyrolean and German immigrants founded the capital of Pozuzo (Pozuzo Centro) in 1859, when they eventually arrived at their destination after a long and exhausting two-year journey from Lima (Figure 3). A second group arrived in 1868. Due to the difficult travelling conditions, the number of people in both groups dropped. The Peruvian state planned to connect the Pacific Ocean to the Atlantic with a direct route and invited Germans and Tyroleans to colonise the land leading up to the Amazon, but this plan was dropped due to the construction of the Panama Canal [58]. The planned road to Pozuzo for 1858 was therefore not built until 1974 which meant that until that time the closest towns could only be reached following a three-day walk. Their isolation meant the migrants endured mainly subsistence living and were therefore able to preserve their costumes and language [58-60]. After the road was built, Peruvian settlers moved to Pozuzo, improvements were made to education and the infrastructure, and cattle’s breeding was established. Increased migration from other parts of Peru led to decreased use of the German “Tiroles” dialect and more intermarrying between “colonists” and Peruvians. Contact with Tyrol was re-established from the 1930s and intensified from the 1970s, which included ongoing financial assistance from Tyrol and Germany [61]. The district of Pozuzo now has a population of 7,760, of whom just 1,038 live in urban areas [62]. About one third of them are descendents of Tyrolean and German colonists [63]. Farming (mainly cattle) is still hugely important for generating income for the people of Pozuzo. The overall health status of the Peruvian population is poor compared with other countries in Latin America. Child mortality rates are high within highland communities and life expectancy in 2009 was 74 for women and 77 for men [64]. In rural areas and small towns like Pozuzo, healthcare services are limited, while in major cities healthcare is more widely available and considered adequate. Thus a large proportion of Peruvians have inadequate access to health services. A “Basic Health-for-All Programme” was introduced into the Peruvian healthcare system by the MINSA (Ministerio de Salud). The programme was aimed at strengthening the health services available to all Peruvian citizens and providing them with access to publicly-run health services.

**Figure 4:**
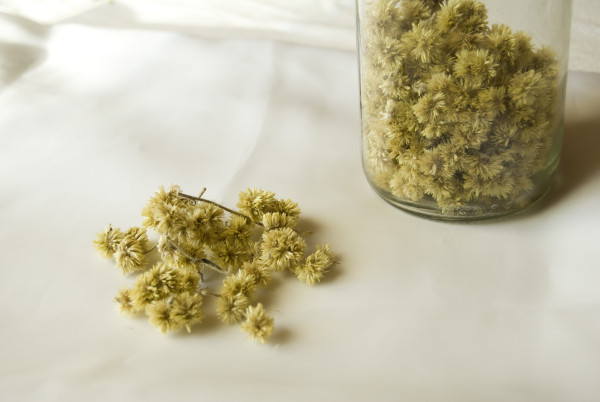
**Dried flowers of “Macela” *(Achyrocline satureioides) *collected by a Tyrolean informant (Photo: Elisabeth Kuhn).**

Since Pozuzian people are quite isolated due to the lack of transport, the first settlers had no medical health care system until the 1940s when the first medical doctor opened his practice in Pozuzo. Before that settlers relied on the use of local medical plants which they learned about from the local indigenous people. A regular clinic was only established in Pozuzo the 1970s and a fully developed clinic, built with the financial support of Austrian donors, was only established in 2004 [61]. Biomedical facilities are provided in the local clinic and the local pharmacies mainly offer pharmaceutical products. Medicinal plants and products are mainly collected in the rainforest or grown in people’s gardens (Figure 4). Although local medicinal plants are still used in household remedies, they have to compete with the exponential increase in modern biomedical facilities. A local indigenous healer with indigenous medical knowledge lives in the area and is consulted by some of the habitants. There are few regions in the world where biological diversity is greater than in the Peruvian Amazon. According to estimates, 8% of the total number of the world’s plant species are found in the region around Pozuzo [65]. For centuries, indigenous peoples have been using plants for healing purposes. Only 1% has been validated so far from a pharmacological or phytochemical point of view and despite its unique plant diversity, few pharmaceutical ingredients have reached the markets in industrialised countries [66].

### Sample

The fieldwork was conducted simultaneously by the first, second and third author in April 2008 in the following Tyrol (T) areas in Austria: Wildschönau in the Lower Inn Valley, Silz in the Upper Inn Valley and Eastern Tyrol. Five respondents were interviewed in each district. Field research was undertaken between June 2008 and December 2008 in Australia (A) in the federal states of New South Wales (Sydney), Queensland (Brisbane and the Gold Coast), South Australia (Adelaide) and Victoria (Beechworth and Melbourne), in Brazil (B) (Treze Tílias) and in Peru (P) (Pozuzo). Prior to field research, initial contact for a first sample was made through people involved in Tyrolean emigration in order to arrange logistics and the organisation of field research in all study areas. The first sample was complemented by snowball sampling. Eligibility criteria for the sample included a minimum age of 18, being of Tyrolean descent (first, second or third generation) and permanently resident in the area of migration. In all, 65 people (A: 20; T, B and P: 15) aged from 26 to 95 (average age: T: 55, stdev 18.5; A: 53, stdev 18.06; B: 63, stdev 18.39; P: 48, stdev 11.87) were interviewed. Endeavours were made to keep the sample gender evenly distributed (A: 55% male / 45% female; B: 47% male / 53% female; P: 53% male / 47% female; T: 47% male / 53% female). In Australia two respondents were interviewed in English, in Brazil nine interviews were conducted in Portuguese and in Peru 14 interviews were held in Spanish. All other interviews were in German. Informants in Australia were all born in Austria, in Brazil informants were first, second and third generation and in Peru all respondents were at least third generation.

### Data acquisition

Prior to the interviews all informants received formal letters explaining the content of the project and assuring them that any data would be used confidentially. Oral informed consent was obtained from the informants for publication of the collected data and any accompanying images. Successive free lists were accomplished with informants to reveal the local plant names of the area’s plants used for medicinal purposes to provide data for further analysis [67,68]. The free-listing question was “Please list all the medicinal plants you know”. After the first listing they were asked: “Is there anything else that comes to mind?” Interviewees will frequently forget to mention a certain taxon because free lists tend only to reflect terms in a respondent’s active vocabulary or they intentionally omit items they know. Therefore an attempt was made to maximise free-list output by repeating the free-listing question. Finally, the free-list items already mentioned were read out and the informants asked again for further quotes. After the free lists were made, the specific medicinal use of each plant given was requested by asking “When and how do you use the plant?”

Then socio-demographic data (name, address, sex, age, place of birth, mother tongue, year of migration, parentage) were recorded. Interview notes were written on prepared forms and all interviews were audio recorded (Olympus Digital Voice Recorder DS-30). After the free lists, informants were asked if they would show their gardens at home, if they had one, so that the plants listed could be documented by taking photographs. The collected socio-demographic data, interview data and related plant data were stored on an MS Access database.

### Plant identification

Although it is obligatory in any botanical research to collect voucher specimens and deposit them in an internationally accessible herbarium, as well as have them professionally identified [69-71] no voucher specimens were taken owing to the organisational difficulties of obtaining plant collection permits for the research sites. As an alternative, detailed photographs were taken of the medicinal plants (the entire plant, stems, bark, leaves, inflorescences, infructescences and exudates) cited in the free list or the product packages of medicinal plants already processed (*e.g.* tea bags, homeopathic products), including their scientific names. Plant photographs were identified by Rodolfo Vásquez M. (researcher at the Jardín Botánico de Missouri, Oxapampa, Pasco, Peru), Fernando Witting Schaus (forestry engineer at the Universidad Nacional Agraria La Molina, Lima, Peru), Osmar do Santos Ribas (curator of the Museu Botânico Municipal herbarium, Curitiba, Brazil) and Prof. Dr. Valdely Ferreira Kinupp (curator of the Instituto Federal de Educação, Ciência e Tecnologia do Amazonas herbarium, Manaus, Brazil) and by specialists from the Plant Identification and Botanical Information Service at the National Herbarium of New South Wales. For plants that were mentioned but could not be photographed because they were not available, were only used in the country of origin or for which data could not be acquired in the growing season, scientific plant names were generated through their local name by literature review [65,66,72-83] as well as by the use of specific websites [84,85]. There is awareness of the fact that the credibility of plant identification through a comparison of literature is in some doubt since confusion of local names with official names when looking them up in botanical literature can lead to errors in plant identification [71]. All scientific plant names were checked through the Missouri Botanical Garden database [86]. Field research was supplemented by taking detailed pictures of medicinal plants or products containing the plants mentioned in the free lists and found in botanical gardens, pharmacies, markets and herb shops.

### Data analysis

As a first step, the free lists were revised for spelling and language. If plant names were given in German as well as in the local language, the name in the local language was chosen for free list analysis. A citation of a medicinal plant in the free list was considered as one single item. Each item corresponds to a specific plant taxon (genus or species level). The free lists were analysed in relation to the items listed and the respondent’s characteristics using ANTHROPAC 4.0 for Windows [67,87]. Calculations were made of (1) the percentage of people who mentioned each item (Resp%), (2) the frequency of mentions per item (F), (3) the average rank of the order in which each item was mentioned (Av rank) and (4) the salience(s) which accounts for frequency and the average rank of items mentioned in the respondent’s list. S is calculated by the formula S = {∑[(L_i_ – R_i_ + 1) / L_i_}/N, where S is the salience of an individual item, L_i_ is the length of an individual list and R_i_ is the rank of the item in that list. Scores range from 1 (maximal salience: first item on every list) to 0. The minimum, maximum and average number of items listed per field site was determined in relation to the respondents [32,67,87]. The analysis of free lists revealed the theoretical knowledge of the plant taxa mentioned. However no evaluations of the plant taxon’s practical importance or significance can be drawn from this since it is possible that a plant taxon scores high in cultural value if people in a culture can name the plant taxon, but low in practical value if people rarely use the plant taxon [88]. After free-list analyses for each field site, the aggregated free lists were compared between field sites using the scientific plant names generated.

#### Agreement indices based on free-listing interviews

One important tool for measuring the distribution of knowledge of medicinal plants is the cultural consensus analysis [67,89]. However, consensus analysis can only be conducted with categorical-type response data (true-false, multiple choice or fill-in-the-blank-question formats). Free-list data cannot be used for consensus analysis since more than one answer for each free-list question is given in free lists [90]. Therefore the proportion of agreement (PA) index [91] based on quantitative data was used. PA measures agreement on the respective domain among respondents through the ratio of the shared items of two respondents to all items listed by two respondents [91]. For example informant 1 listed seven medicinal plants and informant 2 mentioned four. Of these, two plants were mentioned by both respondents and so the PA of the pair of informants is calculated as 2/9. Adding the individual percentages of agreement of one respondent with every other respondent and dividing this sum by the number of respondents produces the overall agreement (OA) of one respondent with every other respondent. Finally, adding together each respondent’s OA and dividing this sum by the number of respondents produces the overall agreement of a free list (OAF). PA is basically similar to consensus analysis, since both indices calculate agreement through the proportion of identical answers between individuals. However for consensus analysis, all respondents are asked the same number of questions while in free-list data collection all respondents list different numbers of items. In consensus analysis all questions are considered for analysis, whereas for PA only those items listed by both respondents are considered for analysis. The items listed by other respondents are left out of the analysis. Hence while in consensus analysis respondents are compared through “matches”, the proportion of agreement (PA) index compares respondents through “positive matches” [92]. An additional difference between consensus analysis and PA is that for PA, the answer does not need to be corrected for guessing, since guessing is not an issue in free listing. PA can be used to measure the extent of agreement among informants and the extent of individuals’ cultural knowledge [91]. Consequently informants who agree with all other informants to a greater percentage can be assumed to have more cultural competence [93]. However, the PA index cannot be used to investigate what information is common knowledge among individuals in a cultural context. In order to reveal a kind of cultural agreement about the items people list and the people who list them, free-list analysis (frequency, average rank and Smith’s salience) [89] is an adequate method.

The single-mentioned (SM) items index was developed by the authors and consists of the ratio of single-mentioned items per field site to the total number of items listed per field site. “Single-mentioned items” are defined as items that are listed in one field site only once. It can be contested that these items belong to the cultural domain of medicinal plants. In contrast, when an item was listed at least twice in an investigation area, minimum agreement is evident about the extent to which this item belongs to the domain. The ratio of single-mentioned items to the total number of items listed in one field site therefore compares the number of contested items to the number of items that definitely belong to the domain investigated. The higher this ratio, the more single-mentioned items are listed in the field site and the higher the disagreement between interviewees about which items belong to the domain. Hence, the single-mentioned items index indicates the level of agreement among respondents in one field site.

#### Cultural importance of plant use value, relative frequency of citation, informant agreement ratio

Different indices were selected to reveal the cultural importance of medicinal plant use and then compared to provide a better understanding of the pattern of medicinal plant knowledge among Tyroleans and Tyrolean migrants and their descendants. The indication of claimed uses remained mostly generic and indefinite (*e.g.* “cleansing, digestive, soothing, relaxing”) which is common in ethnobotany and can be regarded as indicative of genuine information [94].

The number of use reports (UR) provides basic information on the intra-cultural importance of the different plant taxa [95]. Each plant taxon use (t_u_) mentioned by an informant (i) in the use category (u) is considered a “single-use report”. Phillips and Gentry [96] developed the use value (UV) index, a quantitative method that demonstrates the relative importance of plant taxa known locally. It is calculated using the following formula:

(1) $$UV=\sum U_{i}/N$$

U_i_is the number of different uses mentioned by each informant (i) and N is the total number of informants for each study site interviewed for the survey.

To measure the agreement among informants concerning which plants to use for specific use categories, Trotter and Logan’s “informant agreement ratio” (IAR), which is widely used in comparative ethnobotanical studies [97-99] was applied.

The citations of plant uses were grouped into 22 use categories (u) following the economic botany data collection standard [100] as follows: circulatory system disorders, digestive system disorders, endocrine system disorders, genitourinary system disorders, immune system disorders, infections, inflammation, injuries, menstruation/pregnancy/birth/puerperium/menopausal, mental disorders, metabolic system disorders, muscular-skeletal system disorders, nervous system disorders, nutritional disorders, pain, poisonings, respiratory system disorders, sensory system disorders, skin/subcutaneous cellular tissue disorders and unspecified medicinal disorders. The categories ‘cancer’ and ‘others’ were added to the categories of the economic botany data collection standard. Ailments mentioned for the categories are listed in Table 1.

**Table 1:** The 22 categories of use comprising the ailments mentioned by informants in the research areas

	**Ailments**			
**Categories of use**	**Tyrol (n = 15)**	**Australia (n = 20)**	**Brazil (n = 15)**	**Peru (n = 15)**
**Cancer**	-	-	Unspecified cancer	Unspecified cancer
**Circulatory system disorders**	apoplectic stroke, atherosclerosis, blood circulation, blood cleansing, blood pressure, cardio-vascular system, heart	blood cleansing, blood circulation, high blood pressure	anaemia, atherosclerosis, blood circulation blood cleansing, blood poisoning, blood pressure, heart, thrombosis	blood cleansing, blood pressure, cardiovascular system, heart
**Digestive system disorders**	constipation, diarrhoea, digestive, flatulence, laxative, gall bladder, gastrospasm, liver, stomach ache, intestines	diarrhoea, digestive, laxative, stomach ache	stomach ache, cardialgia, diarrhoea, digestion, gastric ulcer, liver pain, intestines, liver, stomach	abstergent, colic, diarrhoea, digestion, gastritis, intestines, liver pain, stomach, flatulence
**Endocrine system disorders**	-	-	diabetes, cholesterol	diabetes, cholesterol, struma
**Genitourinary system disorders**	bladder infection, kidney problems, genitourinary system disorders	bladder infection	bladder infection, diuretic, kidney problems	bladder infection, diuretic, genitourinary system, inflammation of genitals, kidney disorders, kidney stones, prostate
**Immune system disorders**	immune system, lymphatic system	immune system	-	-
**Infections**	anti-bacterial, fever, influenza	cold sores, dermatophytes, disinfection, fever, antipyretic, influenza , insect bites, malaria	antibiotic, fever, influenza, insect bites	antibacterial, antibiotic, dermatophytes, fever, fungal infection, germ killing , insect bites, parasites
**Inflammation**	inflammation	anti-inflammatory	anti-inflammatory	anti-inflammatory
**Injuries**	disinfection, haemostatis, wounds	wounds	injuries, wounds	cuts, wounds
**Menstruation/pregnancy/birth/puerperium/menopausal disorders**	breast feeding, menstrual disorders, menopausal disorders, pregnancy	female disorders, menstrual disorders	abortion	female disorder, menstrual disorders, pregnancy
**Mental disorders**	-	anxiety	-	antidepressant
**Metabolic disorders**	purification	-	weight reduction	anti-oxidant, anti-sweating, weight reduction
**Muscular-skeletal system disorders**	articular gout, aches, lumbago, bone fractures, rheumatism, cramps, rheumatism, joints, sore muscles	joint pain	joint pain	arthritis, bone fractures, joint pain, rheumatism, bone fractures
**Nervous system disorders**	calmative, depression, nervousness, relaxation, sedative	calmative, relaxation, sedative, sleeping	calmative, relaxation, sedative	calmative, relaxation, sedative, sleeping
**Nutritional disorders**	drink, food, condiment, kitchen herb, spice	drink, food, condiment, kitchen herb, spice	drink, kitchen herb, food, spice	anti-oxidant, food, condiment, kitchen herb, spice
**Others**	charm, fragrance, fumigant, fertiliser, moths, ornamental, veterinary use	charm, bugs	veterinary use, not nominated	others
**Pain**	headache, pain relief	headache, toothache	-	pain relief, headache , toothache
**Poisonings**	-	-	-	snake bite
**Respiratory system disorders**	bronchitis, chest, cold, cough, expectorant, gargle, lungs, sore throat	bronchitis , chest, cold, cough, respiratory system, sore throat	cold, cough, influenza, sore throat	asthma, bronchitis, cold, cough, lungs, sore throat
**Sensory system disorders**	eyes, earache	eyes	sinusitis	eyes
**Skin/subcutaneous cellular tissue disorders**	abscess, bruise, burns, hair care, skin care, oral mucosa, burns, warts, zoster, insect bites	burns, bruises, dandruff, dermatitis, eczema, insect bites, hair tonic, massage, skin care, sunburn	hair care, bruises, sunburn	allergies, cicatrices, hair care, skin care, swellings, turgor
**Unspecified medicinal disorders**	bugs, cleansing spirit, first aid remedy, forgetfulness, universal remedy, revitalising, wellbeing	feeling sick, energising , universal remedy, wellbeing, revitalising	tonic, universal remedy, wellbeing	fatigue

The IAR for each use category in the four countries of investigation were calculated using the following formula:

(2) $$IAR=n_{\mathrm{ur}}-n_{t}\cdot /n_{\mathrm{ur}}-1.$$

The informant agreement ratio compares the number of mentions in each use category (n_ur_) and the total number of taxa (n_t_) used in each use category. The values for the factor range from 0 to 1: a higher value indicates agreement among informants and a well-defined medicinal plant tradition [98] since a high value indicates that relatively few taxa are used by most of the people. It is assumed that medicinal use categories with most use records show the most prevalent and common health problems that are treated by the use of medicinal plants.

### Inductive statistics

Correlation analyses (Spearman Rank Coefficient) were performed between socio-demographic variables (age, sex) and the length of free lists and PA. Mann–Whitney tests were conducted using the length of free lists, the proportion of agreement (PA) and use value (UV) as dependent variables and the countries of investigation as independent variables. Inductive statistics were conducted in SPSS16. A one-tailed paired t-test was used to determine whether the average value of the informant agreement ratio (IAR) of Tyrolean migrants and their descendants was not significantly greater than that for people living in Tyrol. Several use categories with undefined IAR were not included in the calculation of the *p* value for the paired t-test. Furthermore, use categories that were not applicable in a studied group of the four countries were not included in the analyses [99]. Therefore the categories included were circulatory system disorders, digestive system disorders, genitourinary system disorders, infections, injuries, nervous system disorders, nutritional disorders, respiratory system disorders, sensory system disorders and skin/subcutaneous cellular tissue disorders. A critical point of the study is the small number of informants, therefore the results of the quantitative analysis are not as reliable from a statistical point of view as they might be and can be regarded as a pilot methodology for providing possible indications for further studies involving a larger number of informants.

## Results

Table 2 provides descriptive statistics of free lists and plant use on medicinal plants in all areas of investigation. Table 3 lists the 15 most salient medicinal plant taxa for each country by vernacular names (as reported by informants), scientific name (genus and species level), along with their botanical families, claimed uses, basic values (respondent percentage, frequency of mention, number of use reports, number of different uses), indices (salience, use value) and their ranking based on the indices. Table 4 shows Informant’s agreement percentages (%) in free lists in all countries. Table 5 illustrates medicinal plant use indices according to use categories (number of taxa in each category, number of use reports, number of use reports percentages, informant agreement ratio) in the different research areas. Table 1 illustrates the 22 categories of use comprising the ailments mentioned by informants in the research areas.

**Table 2:** Descriptive statistics of free lists and plant use on medicinal plant taxa in Tyrol (n = 15), Australia (n = 20), Brazil (n = 16) and Peru (n = 15)*

**Country**	**No of Int**	**Cit total**	**Avg no of cit per int**	**Std. dev. (Avg no of cit per int)**	**Plant taxa total**	**Use cit total**	**No of med cat**
**Tyrol**	15	486	32	19.75	164	552	18
**Australia**	20	193	10	6.24	87	200	16
**Brazil**	15	157	10	4.88	84	180	18
**Peru**	15	303	20	14.05	134	354	21
**Total**	65	1.139	-	-	-	1.286	-

*Coding: No of int: number of interviews; Cit total: total number of citations made by informants; Avg no of cit per int: average number of citations per interview; Std. Dev. (Avg No of cit per Int: Standard deviation (average number of citations listed per interview), Plant taxa total: number of different plant taxa listed; Use cit total: total number of use citations made by informants; No of med cat: number of applied medicinal categories.

**Table 3:** Evaluation of the 15 most salient plant taxa mentioned in free lists, following the Smith’s salience (S) value and their stated uses in Tyrol, Australia, Brazil and Peru

**Country**	**Vernacular name**	**Scientific name**	**Plant family**	**Claimed uses**	**Basic values**				**Indices**		**Ranking**	
					**Resp%**	**F**	**UR**	**NU**	**S**	**UV**	**S**	**UV**
**Tyrol**	Brennessel	*Urtica dioica* L.	Urticaceae	blood cleansing, iron source, food, genitourinary system, hair care, prostate, vitamin provider	80	12	11	7	0.608	0.33	1	4
	Arnika	*Arnica montana* L.	Asteraceae	apoplectic stroke, athlete’s foot, blood circulation, aches, body tension, decongestant, injuries, lumbago, rheumatism, soothing, sore muscles, sprains, stimulant	80	12	12	4	0.6	0.26	2	5
	Frauenmantel	*Alchemilla* spp.	Asteraceae	female disorders, genitourinary system disorders, menopausal problems, wounds	80	12	12	3	0.512	0.2	3	6
	Spitzwegerich	*Plantago lanceolata* L.	Plantaginaceae	animal fodder, cough, croakiness, heart, haemostasis, insect bites, throat, wounds,	80	12	13	4	0.481	0.27	4	5
	Salbei	*Salvia officinalis* L.	Lamiaceae	antibiotic, breastfeeding, cough, fumigant, gargle, kitchen herb, oral mucus, sore throat, stomach	60	9	13	4	0.479	0.27	5	5
	Kamille	*Matricaria chamomilla* L.	Asteraceae	anti-inflammatory, calmative, disinfection, immune system, intestines, relaxation, stomach, universal remedy, wellbeing	80	12	16	6	0.474	0.4	6	3
	Johanniskraut	*Hypericum perforatum* L.	Hypericaceae	antidepressant, burns, depression, ear pain, nervous system, relaxation, sedative, skin and nail care, sun burn, throat ache, well-being	60	9	16	6	0.43	0.4	7	4
	Schafgarbe	*Achillea millefolium* L.	Asteraceae	anticonvulsant, blood cleansing, digestive effect, female disorders, genitourinary system, herbal tonic, influenza, stomach problems, veterinary use	67	10	11	7	0.361	0.47	8	2
	Pfefferminze	*Mentha x piperita* L.	Lamiaceae	aromatic, breastfeeding, cold, cough, headache, increasing blood pressure, sedative, stomach, revitalising	60	9	9	6	0.361	0.4	9	3
	Holunder	*Sambucus nigra* L.	Adoxaceae	bladder infection, cough, drink, fever, fumigant, food, headache, immune system, inflammation, influenza, insect bites, universal remedy, vitamins	67	10	19	8	0.323	0.53	10	1
**Tyrol**	Huflattich	*Tussilago farfara* L.	Asteraceae	blood cleansing, cold, cough	47	7	7	3	0.314	0.2	11	6
	Loewenzahn	*Taraxacum officinale* L.	Asteraceae	cough, blood cleansing, digestion, drink, food, spice, stomach, vitamins	53	8	12	4	0.308	0.27	12	5
	Thymian, Quendel	*Thymus* spp.	Lamiaceae	bronchitis, chest, cold, cosmetic, cough, cramps, fragrance, fumigant, kitchen herb, lungs, relaxation, revitalising	40	6	14	5	0.28	0.33	13	4
	Himmelschlüssel	*Primula veris* L.	Primulaceae	anti-inflammatory, chest, cough, headache, lungs, minerals, throat	53	8	9	5	0.273	0.33	14	4
	Rosmarin	*Rosmarinus officinalis* L.	Lamiaceae	activating, cardio vascular system, drink, kitchen herb, refreshing, stimulating, revitalising, warming	53	8	8	3	0.261	0.2	15	6
**Australia**	Kamille	*Matricaria chamomilla* L.	Asteraceae	calmative, cold, cold sores, eye cleaning, nausea, sedative, stomach ache, universal remedy, wellbeing	75	15	16	6	0.551	0.3	1	1
	Arnika	*Arnica montana* L.	Asteraceae	anti-inflammatory, bones, burns, bruises, cleansing, joints, massage, wounds	60	12	12	4	0.475	0.2	2	3
	Salbei	*Salvia officinalis* L.	Lamiaceae	anti-inflammatory, cold, digestion, drink, kitchen herb, stomach, sore throat	40	8	8	4	0.273	0.2	3	3
	Pfefferminze	*Mentha x piperita* L.	Lamiaceae	chest, cooling, digestive, sedative, stomach, throat	50	10	10	4	0.263	0.2	4	3
	Aloe Vera	*Aloe* spp.	Asphodelaceae	bites, burns, cleansing, dermatophytes, insects, herbal tonic, sunburn, wounds	35	7	8	4	0.232	0.2	5	3
	Brennessel	*Urtica dioica* L.	Urticaceae	circulation system, cleansing, food, revitalising	35	7	9	3	0.164	0.15	6	4
	Knoblauch	*Allium sativum* L.	Amaryllidaceae	blood cleansing, cold, spice, universal remedy	35	7	9	5	0.152	0.25	7	2
	Holunder	*Sambucus nigra* L.	Adoxaceae	cold, drink, fever	25	5	6	3	0.146	0.15	8	4
	Johanniskraut	*Hypericum perforatum* L.	Hypericaceae	blood cleansing, fear, panic attack, relaxation, skin care, stress, sunburn	15	3	4	2	0.132	0.1	9	5
	Linde	*Tilia* spp.	Tiliaceae	cold, fever, nerves, relaxing, wellbeing	20	4	4	4	0.122	0.2	10	3
	Spitzwegerich	*Plantago lanceolata* L.	Plantaginaceae	bronchitis, throat	15	3	3	1	0.121	0.05	11	6
**Australia**	Ginger	*Zingiber officinale* Roscoe	Zingiberaceae	cold, immune system, stomach	15	3	3	3	0.114	0.15	12	4
	Hagebutte	*Rosa canina* L.	Rosaceae	calmative, drink, nervous system, refreshment, skin care, wellbeing,	20	4	5	3	0.112	0.15	13	4
	Zwiebel	*Allium cepa* L.	Amaryllidaceae	antipyretic, cold, inflammation, lungs	15	3	3	3	0.101	0.15	14	4
	Wachholder	*Juniperus communis* L.	Cupressaceae	bladder infection, spice	10	2	2	2	0.1	0.1	15	5
**Brazil**	Babosa°	*Aloe arborescens* Mill.	Asphodelaceae	abscess, burns, cancer, cardialgia, digestive effects, hair care, injuries, stomach, sun burns, universal remedy, thrombosis, ulcer, herbal tonic, liver, wooden splinter, wounds	81	13	6	13	0.64	0.4	1	1
		*Aloe variegata* L.										
		*Aloe vera* (L.) Burm. F.										
	Camomila	*Matricaria chamomilla* L.	Asteraceae	digestion, inflammation, influenza, stomach ache, wounds	56	9	10	4	0.415	0.27	2	2
	Macela	*Achyrocline satureioides* (Lam.) DC.	Asteraceae	digestion, high blood pressure, stomach ache, wound cleansing	56	9	10	6	0.347	0.4	3	1
	Carqueja°	Baccharis articulata (Lam.) Pers.	Asteraceae	diuretic, high blood pressure, strain, joint pain, weight reduction	44	7	6	3	0.313	0.2	4	3
		Baccharis trimera (Less.) DC										
	Losna	*Artemisia absinthium* L.	Asteraceae	digestive, headache, itchiness, stomach	31	5	4	1	0.218	0.27	5	5
	Calendula	*Calendula officinalis* L.	Asteraceae	relaxation, wounds	31	5	4	2	0.185	0.13	6	4
	Cipó mil homens	*Aristolochia triangularis* Cham.	Aristolochiaceae	diarrhoea, internal organs, stomach ache	25	4	4	1	0.175	0.06	7	5
	Hortelã	*Mentha* spp.	Lamiaceae	cough, sedative	25	4	4	2	0.173	0.13	8	2
	Alecrim	*Rosmarinus officinalis* L.	Lamiaceae	calmative, heart	19	3	2	2	0.17	0.13	9	4
**Brazil**	Erva cidreira	*Hyptis althaeifolia* Pohl ex Benth.	Lamiaceae	influenza, relaxation, sedative, stomach	19	3	1	1	0.161	0.06	10	5
		*Lippia alba* var. globiflora (L’Hér.) Moldenke	Verbenaceae									
		*Melissa officinalis* L.	Lamiaceae									
	Quebra pedra	*Phyllanthus niruri* L.	Euphorbiaceae	diuretic, kidneys	25	4	3	1	0.16	0.06	11	5
	Urtiga°	*Urtica* spp.	Urticaceae	blood pressure, weight reduction	31	5	4	2	0.143	0.13	12	4
		*Urera* spp.										
	Boldo°	*Plectranthus barbatus* Andrews	Lamiaceae	digestion, stomach	25	4	4	3	0.141	0.2	13	3
		*Plectranthus neochilus* Schltr.										
	Alcachofra	*Cynara scolymus* L.	Asteraceae	high blood pressure, high cholesterol, stomach	25	4	3	3	0.138	0.2	14	3
	Cidreira	*Cymbopogon citratus* (DC.) Stapf	Poaceae	influenza, relaxation, sedative, stomach	19	3	4	3	0.13	0.2	15	3
		*Hyptis althaeifolia* Pohl ex Benth.	Lamiaceae									
		*Lippia alba var.* Globiflora (L‘Hér.) Moldenke	Verbenaceae									
**Peru**	Llanten°	*Plantago* spp.	Plantaginaceae	anti-inflammatory, cicatrices, disinfection, fever, germ-killing, inflammation, stomach, swells, throat	80	1	13	6	0.626	0.4	1	2
	Uña de gato	*Uncaria guianensis* (Aubl.) J.F. Gmel.	Rubiaceae	cancer, cleansing, inflammation, liver, wounds, pain, swells, tumour	67	1	14	5	0.557	0.33	2	3
	Orégano	*Lantana* spp.	Lamiaceae	baby tea, digestive, gastrospasm, stomach, flatulence	67	1	10	1	0.422	0.06	3	7
		*Mentha spicata* L.										
		*Origanum vulgare* L										
	Matico	*Piper* spp.	Piperaceae	anti-inflammatory, bladder, blister, bruises, cicatrices, cold, cough, infection, kidneys	53	8	13	6	0.389	0.4	4	2
	Bejuco chuncho	*Cissus gongylodes* Burch. Ex Baker	Vitaceae	anti-rheumatism, cancer, disinfection, diabetes, gastritis, joint pain, kidney stones, stomach, turgor, pain relief	53	8	10	7	0.375	0.4	5	1
**Peru**	Sangre de Grado	*Croton lechleri* Müll. Arg.	Euphorbiaceae	cancer, cicatrices, gastritis, wounds	47	7	13	4	0.352	0.26	6	4
	Guanábana	*Anona muricata* L.	Annonaceae	cancer, inflammation, turgor	40	6	6	2	0.233	0.13	7	6
	Guayaba	*Psidium guajava* L.	Myrtaceae	diarrhoea, stomach	40	6	7	2	0.229	0.13	8	6
	Muñá°	*Hyptis mutabilis* (Rich.) Briq.	Lamiaceae	cicatrices, cuts, inflammation	27	4	4	2	0.217	0.13	9	6
		*Minthostachys setosa (Briq.) Epling*										
	Cola de Caballo	Equisetum spp.	Equisetaceae	liver, kidneys	40	6	6	2	0.205	0.13	10	6
	Plano°	*Persea caerulea* (Ruiz & Pav.) Mez	Lauraceae	bone fractures, dislocation, inflammation, swelling	33	5	7	4	0.202	0.26	11	4
		*Nectandra reticulata* (Ruiz & Pav.) Mez										
	Zarzaparrilla	*Serjania rubicaulis* Benth. Ex Radlk.	Sapindaceae	antibacterial, blood cleansing, cicatrices, fungal infection, inflammation, swelling	27	4	5	4	0.19	0.26	12	4
	Achiote	*Bixa orellana* L.	Bixaceae	kidneys, prostate, wounds	20	3	3	2	0.173	0.13	13	6
	Verdolaga	*Portulaca oleracea* L.	Portulacacea	fever, liver, stomach ache	27	4	5	3	0.168	0.2	14	5
	Santa Maria°	*Piper peltatum* L.	Piperaceae	antibiotic, cicatrices	20	3	3	2	0.164	0.13	15	6
		*Piper umbellatum* L.										

Plant ranking is based on each index, the lower the number the higher the rank.

Coding of Variables: Resp% = the percentage of people who mentioned each item; F = the frequency of mentions per item; UR = number of use reports (‘citations’); NU = number of different use reports; S = Smith’s salience, which accounts for frequency and the average rank of items mentioned in the respondent’s list; UV = Use value index; RFC = relative frequency of citation index; ° listed plant item applies to more than one scientific plant taxon.

**Table 4:** Informant’s agreement percentages (%) in free lists (n = 65) in all areas of investigation

**Country**	**Proportion of agreement%**	**Single mentioned plant taxa%**	**Plant taxa mentioned only in the country%**	**Plant taxa also listed in Tyrol%**
**Tyrol**	17	53	20.1	-
**Australia**	11.9	62	4.4	26
**Brazil**	11.2	63	7.7	16
**Peru**	12.2	62	20.3	5.7

**Table 5:** Medicinal plant use indices according to use categories in the different research areas

	**Tyrol**				**Australia**				**Brazil**				**Peru**			
**Categories of use**	**N**_**t**_	**N**_**UR**_	**N**_**UR**_**%**	**IAR**	**N**_**t**_	**N**_**UR**_	**N**_**UR**_**%**	**IAR**	**N**_**t**_	**N**_**UR**_	**N**_**UR**_**%**	**IAR**	**N**_**t**_	**N**_**UR**_	**N**_**UR**_**%**	**IAR**
**Cancer**	0	0	-	N/A	0	0	-	N/A	3	4	2,2	0,333	10	22	6,2	0,571
**Circulatory system disorders**	25	38	6,9	0,351	5	11	5,5	0,6	17	22	12	0,238	13	14	3,9	0,077
**Digestive system disorders**	41	71	13	0,429	15	24	12	0,391	29	63	35	0,548	65	98	27	0,340
**Endocrine system disorders**	0	0	0	N/A	0	0	0	N/A	3	3	1,7	0	11	13	3,6	0,166
**Genitourinary system disorders**	15	24	4,3	0,391	4	4	2	0	7	11	6,1	0,4	18	32	9	0,451
**Immune system disorders**	6	7	1,3	0,167	3	4	2	0,333	0	0	0	N/A	3	3	0,8	0
**Infections**	11	14	2,5	0,231	10	12	6	0,181	5	7	3,9	0,333	23	29	8,1	0,214
**Inflammation**	3	4	0,7	0,333	4	7	3,5	0,5	1	1	0,6	UND	17	28	7,8	0,407
**Injuries**	12	20	3,6	0,421	3	3	1,5	0	9	21	12	0,6	10	10	2,8	0
**Menstruation/pregnancy/birth/puerperium/ menopausal disorders**	6	16	2,9	0,667	3	3	1,5	0	1	1	0,6	UND	3	3	0,8	0
**Mental disorders**	0	0	-	N/A	1	1	0,5	UND	0	0	0	N/A	1	1	0,3	UND
**Metabolic disorders**	3	3	0,5	0	0	0	0	N/A	1	2	1,1	1	3	3	0,8	0
**Muscular-skeletal system disorders**	9	22	4	0,619	1	4	2	1	1	1	0,6	UND	6	11	3,1	0,5
**Nervous system disorders**	12	28	5,1	0,593	12	18	9	0,353	8	9	5	0,125	7	8	2,2	0,143
**Nutritional disorders**	56	107	19	0,481	25	32	16	0,226	6	6	3,3	0	10	14	3,9	0,307
**Others**	27	37	6,7	0,278	3	3	1,5	N/A	5	5	2,8	0	1	1	0,3	UND
**Pain**	4	4	0,7	0	2	3	1,5	0,5	0	0	0	N/A	6	6	1,7	0
**Poisonings**	0	0	-	N/A	0	0	0	N/A	0	0	0	N/A	2	2	0,6	0
**Respiratory system disorders**	37	100	18	0,636	18	32	16	0,452	12	14	7,8	0,154	16	20	5,6	0,211
**Sensory system disorders**	6	7	1,3	0,167	1	1	0,5	UND	2	2	1,1	0	2	2	0,6	0
**Skin/subcutaneous cellular tissue disorders**	14	24	4,3	0,434	11	21	10,5	0,5	2	3	1,7	0,5	20	36	10	0,457
**Unspecified medicinal disorders**	20	26	4,7	0,24	9	17	8,5	0,5	3	5	2,8	0,5	1	1	0,3	UND
**Total**	**307**	**552**	**100**	**-**	**130**	**200**	**100**	**-**	**115**	**180**	**100**	**-**	**248**	**357**	**100**	**-**

Coding: N_t_ = Number of taxa in each category; N_UR_ = Number of use reports; N_UR_% = Number of use reports percentages; IAR = Informant Agreement Ratio.

All respondents (n = 65) were able to answer the free-list question and 1.139 citations were recorded in total (Table 2). There is no significant correlation (Spearman) between the sex or age of respondents and the length of free lists in any of the countries investigated. We recorded 1.286 use citations for 744 different uses belonging to 22 different categories of use. The length of the free list in Tyrol differs significantly from the migrants’ countries (A: p < 0.001; B: p < 0.001, P: p = 0.026). Other than Tyrol, knowledge of medicinal plants was most developed in Peru. The length of free list differs significantly from Australia (p = 0.002) and Brazil (p = 0.029) as indicated by the Mann–Whitney test. In Australia and Brazil the length of the free list is similar (p = 0.366). Use values are significantly different between Tyrol and Australia (p < 0.001) but not between Tyrol and Brazil (p = 0.127) and Tyrol and Peru (p = 0.853).

### Tyrol/Austria

In Tyrol the 15 informants interviewed cited 486 items in the free-list question for medicinal plants (Table 2). The listed items correspond to 164 botanical taxa (Table 2) of which 100 were identified to species and 64 to genus level – belonging to 58 families. The shortest free list consisted of ten items, while the longest had 79 items listed (Table 2). On average the Tyrolean respondents listed 32 medicinal plant taxa per interview. In Tyrol 13 medicinal plant taxa were listed by at least half the respondents (Table 3). The plant taxa quoting the highest salience index was *U. dioica* (s = 0.608). This plant is reported to be used for tea preparation, in tinctures and as a vegetable to provide vitamins and iron and support blood cleansing. Use values ranged between 0.07 and 0.53 (mean = 0.1244, stdev = 0.0885, 95% CI: 0.1107 – 0.1380). *Sambucus nigra* had the highest use value, but this extensively used species would be underestimated if the frequency and salience value alone were used as indices for measuring the plant’s importance. It is still very popular to use the flowers to prepare syrup for cold drinks in summer and infusions (colds, fever, sore throat, influenza, bladder infection, immune system) and its berries are processed into jellies and aspics. For external application, the leaves are used as packing to reduce fever. The plant is regarded as a “universal remedy” and is also used in the preparation of a traditional Tyrolean dish called *Holunder Kiachlen* (flowers dipped in a pancake-like dough, fried in oil and served topped with powdered sugar). *A. montana* is used as a tincture for external application in the form of liniments to treat sprains, bruises, muscle aches, rheumatism and injuries (Table 5). Some of the informants still collect the flower buds of the protected plant that grows at high altitudes and prepare tinctures of it. Informants also report using it in the form of ointments and homeopathic globules. In Tyrol 552 different use reports, assigned into 18 medicinal categories, were recorded (Table 5). The most relevant categories were nutritional disorders (19%) and respiratory system disorders (18%) as listed in Table 5. Most plant taxa listed in the nutritional disorders category were used as a condiment or kitchen herb. *Rosmarinus officinalis* and *Taraxacum officinalis* were the most cited plants in this category. Ailments with the most mentions in the respiratory system disorders category were coughs followed by sore throats (Table 5). The most cited plants in this category were *Plantago lanceolata* and *Salvia officinalis.* The highest agreement values were obtained for ailments related to menstruation/pregnancy/birth/puerperium/ menopausal disorders (IAR = 0.667) with *Alchemilla* sp. as the most common plant taxon, followed by respiratory system disorders, where the most cited plant taxon was *Salvia* officinalis (Table 5).

### Sydney, Gold Coast, Melbourne/Australia

In Australia respondents (n = 20) listed 193 items altogether. The listed items correspond to 87 different botanical taxa (Table 2) - of which 58 were identified to species level and 29 to genus level – belonging to 41 different families. The shortest free list was completed with three items, while the longest list had 31 items. On average, the Australian respondents listed ten items per interview (Table 2). Table 3 lists the most salient plant species. The first three are also listed by half the respondents. Use values ranged from 0.05 to 0.3 (mean = 0.0770, stdev = 0.0533, 95% CI 0.0657 – 0.0884). *Matricaria chamomilla* has the highest use value. The popular plant is only bought in teabags in the supermarket and used to cure colds and stomach aches and is preferably used as a calmative and for general wellbeing. *Arncia montana* is not cultivated in Australia and is therefore only available as a homeopathic product. The tincture is sourced from Germany and France and processed by local companies. One Tyrolean women reported bringing in the tincture from Tyrol after visiting her family there. In Australia 200 use reports for 16 medicinal categories were recorded (Table 5). The most relevant category was nutritional disorders (16%) wherein most plant taxa were used as spices, with the most relevant plants in the category being *Allium sativum* and *Petroselinum crispum* (Table 3). The respiratory system disorders category was frequently mentioned in Australia (16%) (Table 5). Colds and sore throats were the most mentioned ailments of respiratory system disorders, with *Matricaria camomilla* and *Salvia officinalis* the plants most often cited for treating claimed ailments in this category (Table 5). This might be due to the fact that long winters in Tyrol cause many ailments of the respiratory system and although migrants in Australia no longer face lengthy winters, they still remember related uses from their childhood in their home country. All plant taxa named in this category in Australia (*Abies* sp., *Allium sativum*, *Althaea* spp. *Matricaria chamomilla*, *Mentha. x piperita*, *Picea abies*, *Pinus mugo*, *Plantago lanceolato*, *Salvia offiicinalis*, *Sambucus nigra*, *Tilia* spp., *Tussilago. farfara*, *Verascum* spp.) were also named in Tyrol, except for *Backhousia citriodora*, *Eucalyptus* spp., *Zingiber officinale.* These three plant taxa grow in Australia but not in Austria and have been integrated into the medicinal plant knowledge of Tyroleans in Australia.

**Figure 5:**
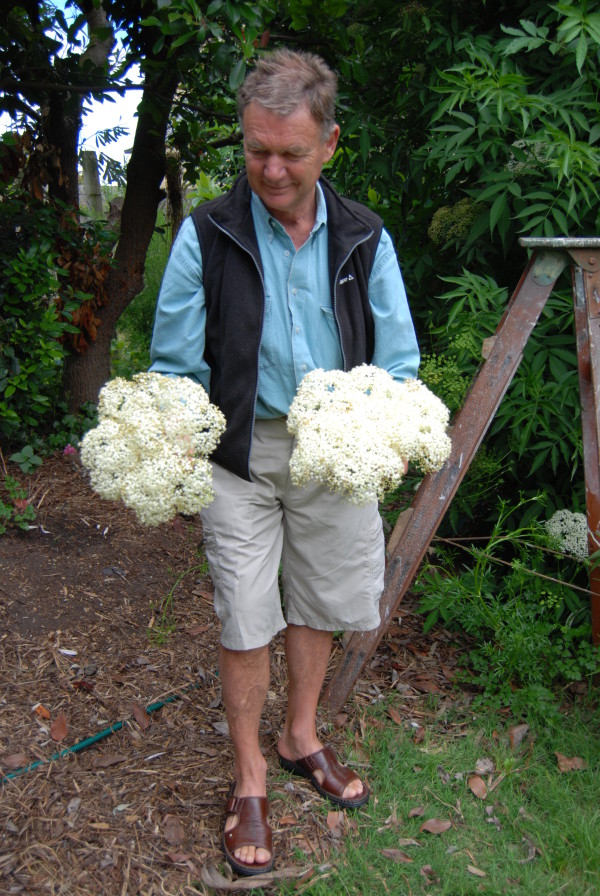
***Sambucus nigra *in the garden of a Tyrolean informant in Australia (Photo: Heidemarie Pirker).**

There was full agreement on the use of *Arnica montana* in the muscular disorders category (IAR = 1) in Australia (Table 5). This category was followed by circulatory system disorders (IAR = 0.6) with *Urtica dioica* as the most cited plant. The number of use reports and use values (p < 0.001) are lower and differ significantly from the other countries of investigation. This might be due to the fact that most Tyrolean migrants live in urban or semi-urban areas with no access to a garden in which they could cultivate the required herbs. It is interesting to note that in the rare cases where people did have a garden, they were very proud of having *Urtica dioica* or *Sambucus nigra* (Figure 5) growing in their garden. Both plants are common and widespread in Tyrol.

### Treze Tílias /Brazil

**Figure 6:**
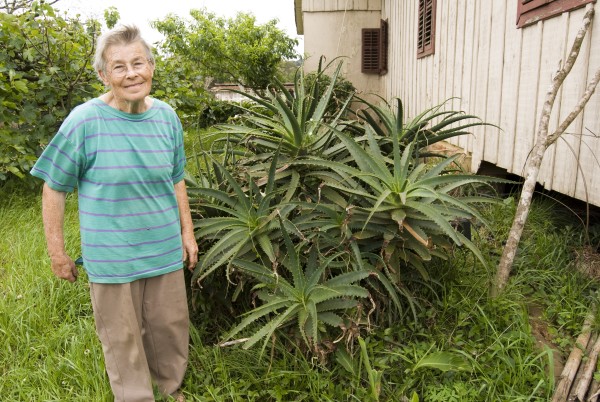
***Aloe *spp. in the garden of a Tyrolean informant in Treze Tílias (Photo: Elisabeth Kuhn).**

In Brazil the respondents (n = 15) listed 157 items altogether and these items correspond to 84 different botanical taxa (Table 2), of which 70 were identified to species level and 14 to genus level, belonging to 57 different families. The shortest free list was completed with four items, while the longest list had 20 items listed (Table 2). On average, ten items were listed per interview (Table 2). The most salient plant taxa mentioned by over half the respondents are listed in Table 3. These taxa also had the highest use values (UV) (Table 5). UV ranged from 0.07 to 0.4 (mean = 0.969, stdev = 0.0623, 95% C 0.0837 – 0.1102). *Aloe spp*. is widely distributed in this area and grows in almost every garden. It is seen as a “universal remedy” among informants. The plant is claimed to cure injuries, burns, wounds, stomach ulcers and digestive problems, as well as prevent cancer. There are many different recipes for its preparation: pulp from the inner leaves is eaten fresh or frozen, and mixed with honey by some informants. *Matricaria chamomilla* grows in almost every garden and is hardly ever bought in a shop. *Achyrocline satureioides* (Figure 4) uses are similar to the uses of *M. chamomilla* (Table 3) although *A. satureioides* is cited more often in use as a bath additive and for the preparation of tea for young children (Figure 4). While *M. chamomilla* can be seen as a ”universal plant”, the distribution of *A. satureioides* is more likely to be limited to South America and has substituted some of the uses attributed to *M. chamomilla* in Tyrol. In Brazil 180 use reports for 18 categories were recorded (Table 5). The gastrointestinal disorders category was the most prevalent in Treze Tílias (35%), with stomach disorders the most frequently mentioned ailments. Some of the plants cited and used by Tyrolean migrants and their descendents help support digestion after a “heavy meal”, which is quite common in Treze Tílias. After the indulgence of “savoury roast pork”, “goulash” or the beloved “churrasco”, a tea made from the leaves of Pneumus boldus, Cynara cardunculus or Mentha spp. is an essential conclusion to the meal. Serious trouble with digestion might even require tea from *Artemisia absinthium* and the fresh pulp of *Aloe* spp. (Figure 6) helps overcome digestive discomforts.

**Figure 7:**
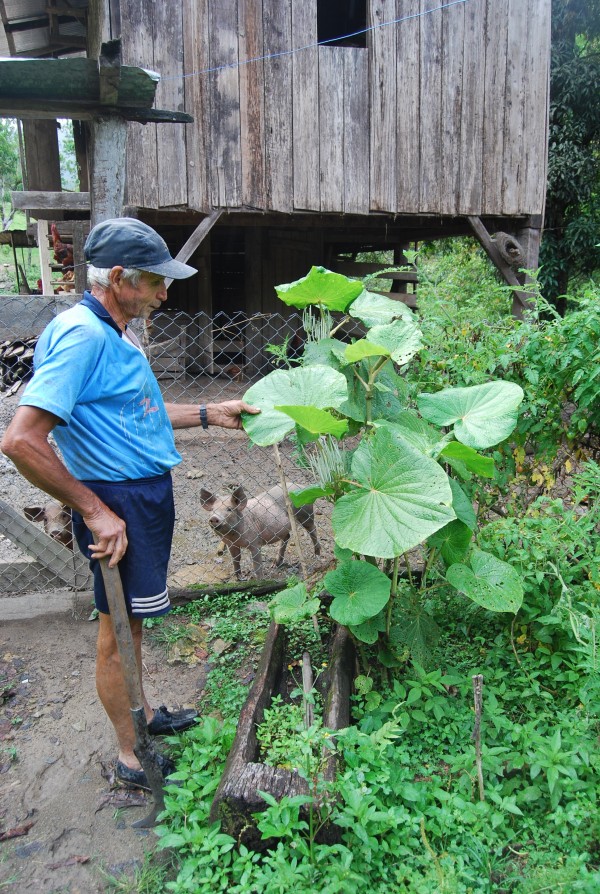
**“Santa Maria” *(Piper spp.) *in the garden of a Tyrolean descendant in Pozuzo (Photo: Ruth Haselmair).**

Agreement on the use of medicinal plants was highest for the metabolic system disorders category (IAR = 1), with *Urera* spp. as the cited plant, followed by injuries (IAR = 0.6) and gastrointestinal disorders (IAR = 0.548), both categories having *Aloe* spp. as the most cited plant taxon (Table 5). During interviews, respondents in Brazil reported that some migrants tried to bring plants traditionally used for healing purposes in Tyrol with them. Not all of them suited the growing conditions in Treze Tílias. One informant remembered that she brought *Allium schoenoprasum* and *Ribes rubrum* to grow in her garden, but the latter could not be cultivated in Treze Tílias due to the growing conditions there. The migrants themselves grow plants such as *Matricaria chamomilla, Aloe spp., Calendula officinalis, Urtica dioica* and many more in their own gardens for use as home remedies for ailments.

### Pozuzo/Peru

In Peru respondents (n = 15) listed 303 items altogether. These items correspond to 134 different botanical taxa (Table 2) – 92 were identified to species level and 36 to genus level –belonging to 57 families, while 6 remain unidentified. The shortest free list was completed with six items, while the longest list was completed with 52 items (Table 2). On average, Peruvian respondents listed 20 items (Table 2). The most salient plant taxa, the first six mentioned in Peru by more than half the respondents, are listed in Table 3.

Use values (UV) ranged between 0.07 and 0.47 (mean = 0.1185, stdev = 0.08115, 95% CI 0.1046 – 0.1323. *Cissus gongylodes reached* the highest UV (Table 3). In Peru 354 use reports for 22 categories were recorded (Table 5). The most relevant medicinal category was gastrointestinal system disorders (27%), with stomach disorders the most frequently mentioned ailment. Plant species mentioned most often in this category were *Psidium guajava*, *Croton lechleri* and *Verbena litoralis*. Agreement on the use of medicinal plants was highest in the cancer category (IAR = 0.571), with *U. guianensis* the most cited plant, followed by skin/subcutaneous cellular tissue disorders (IAR = 0.457) with *C. lechleri* the most cited plant species (Table 5).

### Agreement between informants and an overall comparison

Table 4 shows the percentages of proportion of agreement (PA), single-mentioned (SM) plant taxa among informants in all research areas, other plant taxa mentioned in only one country and plant taxa cited in both the migrants’ country and in Tyrol. The highest proportion of agreement (PA) in free lists was calculated for Tyrol (17%), followed by Peru (12.2%), Australia (11.9%) and Brazil (11.2%). The proportion of agreement differs significantly between informants in Australia and Tyrol (p = 0.001), Brazil and Tyrol (p = 0.001) and Peru and Tyrol (p = 0.001) and is similar between informants among the migrant countries as indicated by the Mann–Whitney test. There are no significant correlations between the sex or age of respondents and the proportion of agreement.

The proportion of single-mentioned botanical taxa was lowest for informants in Tyrol (53% of all taxa listed in Tyrol), followed by Peru (62%), Australia (62%) and Brazil (63%). The number of plant taxa listed only in Tyrol is 91 (20.1%), 92 (20.3%) in Peru, 35 (7.7%) in Brazil and 20 (4.4%) in Australia.

Of all the botanical taxa listed, five (1.1%) were listed in all four investigation countries (*Allium sativum, Artemisia absinthium, Brassica oleracea*, *Matricaria* spp. *and Plantago* spp.). In Australia and Tyrol together, 199 different plant taxa were listed. Of these, 52 (26%) were listed in both regions, while 33 (17%) were listed in Australia only and 114 plant taxa (57%) were listed in Tyrol only. In Treze Tílias and Tyrol, 210 different plant taxa were listed. Of these, 33 plant species (16%) were listed in both regions, while 46 plant species (22%) were listed in Treze Tílias only and 131 plant species (62%) were listed only in Tyrol. In Pozuzo and Tyrol, 278 different plant taxa were listed in total. Of these, 16 plant species (5.7%) were listed in both regions, while 109 plant species (39%) were listed in Pozuzo only and 153 plant species (55%) were listed in Tyrol only.

It is assumed that the use categories with the highest number of mentions were the most common and important among Tyroleans and the migrants. In no investigation area did the use category with the highest number of use mentions match the ones informants agreed on most. For the categories included in the statistics, the average informant agreement ratio (IAR) in Tyrol is significantly higher than in Australia (p = 0.089) and Brazil (p = 0.238) but not Peru (p = 0.019).

## Discussion

The knowledge held by Tyroleans and Tyrolean migrants and their descendants about medicinal plants as it is today is presented approximately 50 years after emigration to Australia, 80 years after emigration to Brazil and 150 years after emigration to Peru. The different timescales offer insights into how transformation processes on traditional knowledge progressively occurred in the host cultures. Tyrolean migrants and their descendants experienced different societal and environmental influences in the course of their migration history. All respondents in all four investigation areas claimed to know and use medicinal plants to treat basic ailments in their day-to-day lives. Although the tendency has been observed in studies for older informants to have greater knowledge of medicinal plants, these findings are not supported by this research [101,102]. Results in research carried out on gender differences within knowledge distribution concerning ethnobotanical knowledge are inconsistent [101-104]. Our findings show that men and women have similar knowledge of medicinal plants in all the countries investigated.

As described before in ethnobotanical studies on this subject [4], processes of continuation and adaptation of medicinal plant knowledge both took place during the migration of people from Tyrol. The length of free lists, number of plant taxa cited, number of use citations, use values (UV) and the proportion of agreement (PA) are highest in Tyrol and the number of single-mentioned (SM) items lowest. These results relate to a well-established tradition of medicinal plant knowledge in the country of departure and indicate that knowledge and use of medicinal plants is most pronounced there. Therefore migration clearly had a corrosive effect on the traditional knowledge of medicinal plants and their applications among migrants as compared to informants living in Tyrol. Reduced ethnomedicinal knowledge among migrant groups compared with their country of origin has been reported before [9,15,21]. Kirsch [26] recognises that local knowledge may be lost when it depends on continued access to specific land and resources. If some resources are unavailable for migrants they will no longer teach their descendants specific knowledge linked to these resources. However, culturally salient species continue to be important after migration [30] and their uses tend to be more resilient towards changes if they are relevant elements of global knowledge [16,22,24]. Cosmopolitan plants (*e.g. Matricaria chamomilla*, *Hypericum perforatum*, *Plantago* spp., *Urtica* spp.) are most likely to be used continuously and this can be explained by the fact that they are regarded as medicinally very effective and are also often easy to access. The plants listed in all four areas of investigation for medicinal purposes (*Allium sativum, Artemisia absinthium, Brassica oleracea*, *Matricaria* spp. *and Plantago* spp.) could be considered such species and therefore used continuously.

Due to the fact that all Tyrolean respondents living in Australia were born and brought up in Tyrol and therefore still remember the medicinal plants and may have experienced their use during childhood consensus on the knowledge of medicinal plant taxa (26%) is highest between Tyrol and Australia. Further, all informants in Australia still return to Austria on a regular basis and therefore probably stay in closer contact with the traditions of their country of birth as compared to the other research areas. The dispersed settlement of Tyrolean emigrants, the increasing influence of urbanisation with mostly no access to a garden and well-established healthcare facilities made the continuation strategy with some new incorporated plants the most obvious one to establish among migrants in Australia. It is striking that out of the 87 medicinal plants named in free listing in Australia, only four (*Eucalyptus spp.*, *Melaleuca alternifolia, Xanthorrhoea spp. and Acacia spp.*) are native to Australia. Although Australian Indigenous people had and still have extensive knowledge of medicinal and native Australian plants, other than *Eucalyptus spp.* and *M. alternifolia* they have failed to enter existing herbal pharmacopoeias [45]. Strict import and quarantine regulations on flowers, plants and plant-related materials have been implemented to protect Australia’s unique nature, hampering the import of plants and plant-related material, and therefore the private import of medicinal plants or products to Australia (*e.g.* herbal tea mixtures or homespun remedies) is negligible. Nevertheless, many herbs in the traditional Tyrolean pharmacopoeia can be obtained from herbalists, naturopaths and health care shops. This mode of acquisition is probably the most dominant here compared to the other areas investigated, where informants mostly source medicinal plants from their own garden and through collection in the wild. Tyroleans in Australia are not in close contact with one other and live more individual lives, so the absence of a network that supports the exchange of knowledge of medicinal herbs might also have a detrimental effect on the use of traditional Tyrolean medicinal herbs, with only a small amount of knowledge of traditional medicinal herbs being passed on to the next generation.

Compared with Latino migrants in New York who continue to import, trade and use herbs to serve their ethnic community in special shops such as botánicas in New York [5,7] or in Amsterdam, where a store specialising in medicinal plants from Suriname offers fresh, dried and frozen medicinal plants from Suriname to the immigrant community [19], this study’s findings show that Tyrolean migrants in both Brazil and Peru adapted to the new flora and related health practices in the host country. Migrant groups’ knowledge of medicinal plants in Brazil and Peru has changed more than it has in Australia. The shared knowledge among Tyroleans in Brazil differs a great deal from the shared knowledge about medicinal plants in Tyrol. Migrants in Brazil faced the influence of migrants from different continents, which led to a mixture of continuation and adaptation processes, although it is difficult to track down the origin of a plant because of an absence of information about the geographic distribution of species growing in both the country of origin and the host country.

Nowadays Tyrolean migrants and their descendants in Treze Tílias have knowledge of medicinal plants known in Brazil and Tyrol. Some plants which were not mentioned during free listing in Tyrol obtained a high score in the Smith’s salience index in the free lists in Brazil, such as *Aloe* spp., *Achyrocline satureioides* or *Baccharis articulata* and *Baccharis trimera.* These plants can be regarded as substituting plants from the Tyrolean pharmacopoeia that were not easily accessible in Brazil. Individual imports of plants and plant products from Austria are negligible since permission is required from the Ministry of Agriculture before they can be imported into the country.

In Peru and Tyrol no plant taxon listed in both countries was mentioned by more than a third of respondents. Therefore it can be assumed that Tyrolean migrants in Peru substituted and replaced most of the traditional medical plants from Tyrol. When Tyrolean migrants finally arrived in the remote area of Pozuzo in Peru, they faced a situation in which they had to build a completely new settlement. Therefore people had to rely on the knowledge of indigenous groups on how to use plants from the rich local flora of the tropical rainforest as they were also confronted by completely different illnesses (*e.g.* malaria, typhus and yellow fever) than those found in their home country. This explains the fact that the percentage of plants only named in Peru was highest and that consensus on medicinal plants with informants in Tyrol was lowest. Out of the ten most salient plants listed by informants in Pozuzo, only *Plantago spp.* is also found among the most salient plants in Tyrol. After three generations the medicinal knowledge of traditional Tyrolean plants has been exchanged almost completely. Many plants from Tyrol have been forgotten over the years as they do not grow in tropical conditions and they were substituted by other plants. New plants for medicating diseases which were unknown until then also became incorporated. Therefore, the circumstances of long-lasting isolation led to the adaptation strategy in Peru. Medical healthcare facilities have now improved and are geared towards conventional medicine, while the use of medicinal plants is starting to play a secondary role as globalisation, increasing industrialisation and the accelerating destruction of botanically rich native ecosystems are challenging the continuation of traditional medicinal health practices. Nevertheless there are still Pozuzian people who collect medicinal plants or cultivate them in their gardens (Figure 7). Use values (p = 0.853) come close to Tyrolean informants, which indicates that there is a strong tradition still alive around the use of medicinal plants. Since people in Pozuzo have begun to recognise the value of the tropical rainforest, with Peru’s flora providing one of the world’s richest sources for plant-based medicines [65,105] and they are very important commercially, there is now increasing interest in specific medicinal plants. One example that has come to light in recent years is “Uña de gato” (*Uncaria guianensis*) which is a powerful medicinal plant thought to provide relief for inflammation and rheumatic diseases [65]. The woody vine that takes its name from the hook-like thorns that grow along it resembling a cat’s claw has a long history of use among the indigenous people of the Amazonian Rainforest [106]. Since “Uña de gato” seems to have positive effects in the curing of cancer, the plant is “booming” on the international pharmaceutical trade market ever since, resulting in it being well known in Pozuzo.

## Conclusions

Tyrolean migrants did not simply adapt to a new medical culture when they migrated to another country. Instead the use of traditional medicinal plants continued concurrently and progressively, some practices were abandoned and new elements were integrated into the medicinal plant knowledge system. We conclude that the main processes that happened in knowledge transformation in all countries were to abandon specific medicinal plants and related practices from the original pharmacopoeia if the plants were neither available nor cultivated in the country of arrival. The social ties between the relatively low numbers of migrants and their country of origin were not constant and strong enough to offer greater scope for the importation of important plants from the home flora. In conclusion, it can be stated that the choice of medicinal plant use decades after Tyroleans’ migration to Australia, Brazil and Peru was greatly influenced by the existing environmental and social conditions in the country of arrival, the predominant healthcare system, the degree of contact with the local population (*e.g.* social networks) and the home country and the opportunities for acquiring plants through importation. Overall, our findings indicate that the medicinal plant tradition in Tyrol and Pozuzo is more clearly defined than among the Tyroleans and their descendants interviewed in Australia and Treze Tílias, although the knowledge in Peru has undergone major transformational shifts compared to Australia and Brazil where the various influences from other migration processes may have had a more dispersing influence on the field of medicinal plants along with an eroding effect on the degree of knowledge.

## Competing interests

The authors declare that they have no competing interests.

## Authors’ contributions

HP, EK, RH and CRV conceived and designed the research. HP, EK and RH carried out field research in Tyrol, with research also being conducted by HP in Australia, EK in Brazil and RH in Peru. HP performed data analysis and drafted the manuscript. CS performed and made a substantial contribution to data analysis (free list, PA, SM, OA-Indices). EK, RH, CS and CRV critically revised the manuscript for intellectual content. All authors read and approved the final manuscript.

## References

[B1] IOM World Migration Report 2010 Geneva: International Organization for Migration

[B2] Pieroni A Vandebroek I Pieroni A, Vandebroek I Introduction Traveling Cultures and Plants. The Ethnobiology and Ethnopharmacy of Human Migrations 2007 Oxford: Berghahn 1 12

[B3] Volpato G Godínez D Beyra A Migration and Ethnobotanical Practices: The Case of Tifey Among Haitian Immigrants in Cuba Hum Ecol 2009 37 1 43 53 10.1007/s10745-008-9211-4

[B4] Medeiros PSG Alencar N Vandebroek I Pieroni A Hanazki N De Albuquerque UP The Use of Medicinal Plants by Migrant People: Adaptation, Maintenance and Replacement Evidence-Based Complementary and Alternative Medicine 2011 in press 10.1155/2012/807452 PMC3216396 22110548

[B5] Balick M Kronenberg F Ososki A Reiff M Fugh-Berman A Bonnie OC Roble M Lohr P Atha D Medicinal plants used by Latino healers for women’s health conditions in New York City Economic Botany 2000 54 3 344 357 10.1007/BF02864786

[B6] Reiff M O’Connor B Kronenberg F Balick MJ Lohr P Roble M Fugh-Berman A Johnson KD Ethnomedicine in the urban environment: Dominican healers in New York City Hum Organ 2003 62 12 26

[B7] Vandebroek I Balick MJ Yukes J Duran L Kronenberg F Wade C Ososki AL Cushman L Lantigua R Mejía M Robineau L Pieroni A, Vandebroek I Use of Medicinal Plants by Dominican Immigrants in New York City for the Treatment of Common Health Conditions: A Comparative Analysis with Literature Data from the Dominican Republic Traveling Cultures and Plants The Ethnobiology and Ethnopharmacy of Human Migrations 2007 Oxford: Berghahn 39 64

[B8] Waldstein A Diaspora and Health? Traditional Medicine and Culture in a Mexican Migrant Community Int Migr 2008 46 5 95 117 10.1111/j.1468-2435.2008.00490.x

[B9] Volpato G Godinez D Beyra A Barreto A Uses of medicinal plants by Haitian immigrants and their descendants in the Province of Camaguey, Cuba J Ethnobiol Ethnomed 2009 5 1 16 10.1186/1746-4269-5-16 19450279 PMC2690575

[B10] Pieroni A Quave C Nebel S Heinrich M Ethnopharmacy of the ethnic Albanians (Arbëreshë) of northern Basilicata, Italy Fitoterapia 2002 73 3 217 241 10.1016/S0367-326X(02)00063-1 12048017

[B11] Pieroni A Dibra B Grishaj G Grishaj I Gjon Maçai S Traditional phytotherapy of the Albanians of Lepushe, Northern Albanian Alps Fitoterapia 2005 76 3–4 379 399 15890470 10.1016/j.fitote.2005.03.015

[B12] Pieroni A Muenz H Akbulut M Baser KHC Durmuskahya C Traditional phytotherapy and trans-cultural pharmacy among Turkish migrants living in Cologne, Germany J Ethnopharmacol 2005 102 1 69 88 10.1016/j.jep.2005.05.018 16002248

[B13] Pieroni A Quave CL Traditional pharmacopoeias and medicines among Albanians and Italians in southern Italy: A comparison J Ethnopharmacol 2005 101 1–3 258 270 15978757 10.1016/j.jep.2005.04.028

[B14] Quave CL Pieroni A Pieroni A, Vandebroek I Traditional Health Care and Food and Medicinal Plant Use among Historic Albanian Migrants and Italians in Lucania, Southern Italy Traveling Cultures and Plants The Ethnobiology and Ethnopharmacy of Human Migrations 2007 Oxford: Berghahn 204 227

[B15] Pieroni A Vandebroek I Traveling Cultures and Plants. The Ethnobiology and Ethnopharmacy of Human Migrations 2007 Oxford: Berghahn

[B16] Ceuterick M Vandebroek I Torry B Pieroni A Cross-cultural adaptation in urban ethnobotany: The Colombian folk pharmacopoeia in London J Ethnopharmacol 2008 120 3 342 359 10.1016/j.jep.2008.09.004 18852036

[B17] Maxia A Lancioni M Balia A Alborghetti R Pieroni A Loi M Medical ethnobotany of the Tabarkins, a Northern Italian (Ligurian) minority in south-western Sardinia Genet Resour Crop Evolution 2008 55 6 911 924 10.1007/s10722-007-9296-4

[B18] van Andel T Westers P Why Surinamese migrants in the Netherlands continue to use medicinal herbs from their home country J Ethnopharmacol 2010 127 3 694 701 10.1016/j.jep.2009.11.033 20004237

[B19] Van Andel T Van TKlooster C Pieroni A, Vandebroek I Medicinal Plant Use by Surinamese Immigrants in Amsterdam, the Netherlands: Results of a Pilot Market Survey Traveling Cultures and Plants The Ethnobiology and Ethnopharmacy of Human Migrations 2007 Oxford: Berghahn 122 145

[B20] Pieroni A Zaman H Ayub STB Pardo de Santayana M, Pieroni A, Rajindra KP My Doctor Doesn’t Understand Why I Use Them’ Herbal and Food Medicines amongst the Bangladeshi Community in West Yorkshire, U.K Ethnobotany in the New Europe People, Health and Wild Plant Resources 2010 New York, NY: Berghahn Books 112 146

[B21] Ceuterick M Vandebroek I Pieroni A Resilience of Andean urban ethnobotanies: A comparison of medicinal plant use among Bolivian and Peruvian migrants in the United Kingdom and in their countries of origin J Ethnopharmacol 2011 136 1 27 54 10.1016/j.jep.2011.03.038 21470576

[B22] Vandebroek I Balick MJ Globalization and Loss of Plant Knowledge: Challenging the Paradigm PLoS One 2012 7 5 e37643 10.1371/journal.pone.0037643 22662184 PMC3360753

[B23] John M Horvath T Arbeitslosigkeit und Auswanderung in Österreich 1919–1937 Auswanderungen aus Österreich von der Mitte des 19. Jahrhunderts bis zur Gegenwart 1996 Austria: Böhlau Verlag Wien 83 110

[B24] Berkes F Colding J Folke C Rediscovery of Traditional Ecological Knowledge as adaptive management Ecol Appl 2000 10 5 1251 1262 10.1890/1051-0761(2000)010[1251:ROTEKA]2.0.CO;2

[B25] Warburton H Martin A Local people’s knowledge in natural resources research Socio-economic Methodologies for Natural Resources Research 1999 Chatham, UK: Natural Resources Institute

[B26] Kirsch S Lost Worlds Curr Anthropol 2001 42 2 167 198 10.1086/320006

[B27] Nesheim I Dhillion S Anne Stølen K What Happens to Traditional Knowledge and Use of Natural Resources When People Migrate? Hum Ecol 2006 34 1 99 131 10.1007/s10745-005-9004-y

[B28] Lee RA Balick MJ Ling DL Sohl F Brosi BJ Raynor W Cultural dynamism and change in Micronesia Economic Botany 2001 55 9 13 10.1007/BF02864542

[B29] Ososki AL Balick MJ Daly DC Pieroni A, Vandebroek I Medicinal Plants and Cultural Variation across Dominican Rural, Urban, and Transnational Landscapes Traveling Cultures and Plants The Ethnobiology and Ethnopharmacy of Human 2007 Oxford: Berghahn 14 39

[B30] Nguyen MT Comparison of food plant knowledge between urban Vietnamese living in Vietnam and Hawaii Economic Botany 2003 57 4 474 480 The New York Botanical Garden Press, Bronx, New York, USA

[B31] Ososki AL Balick MJ Daly DC Pieroni Andrea IV Medicinal Plants and Cultural Variation across Dominican Rural, Urban, and Transnational Landscapes Traveling cultures and plants. The ethnobiology and ethnopharmacy of human migrations 2009 New York: Berghahn Books 14 39

[B32] Vogl CR Vogl-Lukasser B Puri RK Tools and methods for data collection in ethnobotanical studies of homegardens Field methods 2004 16 3 285 306 10.1177/1525822X04266844

[B33] Vogl-Lukasser B Lokales Erfahrungswissen über Pflanzenarten aus Wildsammlung mit Verwendung in der Futterung und als Hausmittel in der Volksheilkunde bei landwirtschaftlichen Nutztieren in Osttirol 2006 Okologischen Landbau: Endbericht. Wien: Inst. f Projektnummer: 1272, GZ 21.210/41-II1/03

[B34] Vogl-Lukasser B Vogl CR Ethnobotanical Research in Homegardens of Small Farmers in the Alpine Region of Osttirol (Austria) Ethnobotany Res Appl 2008 2 2 111 137 Photo Essay 2004 (ISSN 1547–3465)

[B35] Christanell A Vogl-Lukasser B Vogl CR Gütler M Pardo de Santayana M, Pieroni A, Rajindra KP The Cultural Significance of Wild-gathered Plant Species in Kartitsch (Eastern Tyrol, Austria) and the Influence of Socioeconomic Changes on Local Gathering Practices Ethnobotany in the New Europe People, Health and Wild Plant Resources. Volume Studies in environmental anthropology and ethnobiology 2010 New York, NY [u.a.]: Berghahn Books 51 75

[B36] Tirol L Statistisches Handbuch Bundesland Tirol 2009 In Innsbruck: Land Tirol

[B37] Vogl-Lukasser B Erfahrungswissen über Lokalsorten traditioneller Kulturarten in Ost- und Nordtirol. Projekt durchgeführt im Rahmen des INTERREG IIIA Tirol - Sudtirol zur Sicherung pflanzlicher Genressourcen in den Alpen (Gene-Save) und des Projektes 1272, GZ 21.210/41-II1/03 (Teil 2) gefördert vom Land Tirol und dem Lebensministerium (BM:LFUW) 2007 Endbericht. Wien: Inst. f. Okologischen Landbau - Univ. f. Bodenkultur

[B38] Cl B Health care in Austria: Universal access, national health insurance, and private health care JAMA 1993 269 21 2789 2794 10.1001/jama.1993.03500210089039 8492408

[B39] Norst M Austrians and Australia 1988 Sydney: Athena Press

[B40] Jupp J The Australian People 2001 Cambridge: Cambridge University Press

[B41] NAA - National Archives of Australia Applications for Registration by Alien Entering Australia Canberra: Australian Government http://www.naa.gov.au

[B42] Pink B Migration Australia 2007–08 2009 Canberra: Australian Bureau of Statistics

[B43] Orchard AE Orchard AE, Thompson HS A history of systematic botany in Australia Flora of Australia. Introduction. Vol. I 1999 Melbourne: Australian Biological Resources Study/CSIRO 11 103

[B44] Commonwealth Department of Health and Aged Care An Overview of Health Status, Health Care and Public Health in Australia. Occasional Papers Series Number 5 2000 Canberra: Australian

[B45] Wohlmuth H Oliver C Oliver N A Review of the status of western herbal medicine in Australia J Herb Pharmacother 2002 2 2 33 46 10.1080/J157v02n02_04 15277095

[B46] Evans S Joseph Banks and the continuing influence of European colonisation on Australian herbal practice Aust J Med Herbalism 2009 21 3 63 65

[B47] IBGE Censo Populacional 2010 2010 Brazil: Instituto Brasileiro de Geografia e Estatística

[B48] Prutsch U Horvath T, Neyer G Brasilien - Die Suche nach einer neuen Heimat. Die Auswanderung von ÖsterreicherInnen nach Brasilien 1918–1938 Auswanderungen aus Österreich Von der Mitte des 19Jahrhunderts bis zur Gegenwart 1996 Wien Köln Weimar: Böhlau Verlag 111 128

[B49] Reiter M Osl M Humer A 75 Jahre Dreizehnlinden. Treze Tílias 2008 Tirol: Reith im Alpbachtal

[B50] Achrainer K Eisterer K Dreizehnlinden – An Austrian Immigration Settlement in Brazil during the Great Depression Transatlantic relations: Austria and Latin America in the 19th and 20th centuries 2006 Wien: Studien-Verl, Innsbruck 137 161

[B51] Thaler A Die österreichische Kolonie Dreizehnlinden in Brasilien 1934 2 Innsbruck: Selbstverlag der " Österreichischen Auslandssiedelungsgesellschaft

[B52] Ilg K Pioniere in Brasilien durch Bergwelt, Urwald und Steppe erwanderte Volkskunde der deutschsprachigen Siedler in Brasilien und Peru 1972 Innsbruck, Wien [u.a.]: Tyrolia-Verl

[B53] Ilg K Heimat Südamerika Brasilien und Peru; Leistung und Schicksal deutschsprachiger Siedler. 2 1982 Innsbruck, Wien: Tyrolia-Verl

[B54] Reiter M Rampl M Humer A Dreizehnlinden. Österreicher im Urwald 1993 Berenkamp: Schwaz

[B55] Brandão MGL Zanetti NNS Oliveira P Grael CFF Santos ACP Monte-Mór RLM Brazilian medicinal plants described by 19th century European naturalists and in the Official Pharmacopoeia J Ethnopharmacol 2008 120 2 141 148 10.1016/j.jep.2008.08.004 18762237

[B56] Vieira RF Janick J Conservation of medicinal and aromatic plants in Brazil Perspectives on new crops and new uses 1999 VA: ASHS Press, Alexandria

[B57] Brandão MGL Acúrcio FA Montemor RLM Marlière LDP Complementary/Alternative Medicine in Latin America: Use of Herbal Remedies among a Brazilian Metropolitan Area Population J Complement Integr Med 2006 3 1 5

[B58] Schütz-Holzhausen D Der Amazonas. Wanderbilder aus Peru, Bolivia und Nordbrasilien 1895 2 Freiburg im Breisgau: Herdersche Verlagshandlung

[B59] Ilg K Heimat Südamerika: Brasilien und Peru; Leistung und Schicksal deutschsprachiger Siedler 1982 2 Tyrolia-Verlag: Innsbruck-Wien

[B60] Schabus W Pozuzo. Varietätenausgleich und Sprachkontakt in einer deutschen Sprachinsel in Peru Mundart und Name im Sprachkontakt Festschrift für Maria Hornung zum 1990 Mundart und Name im Sprachkontakt Festschrift für Maria Hornung zum 70 VWGÖ: Wien 205 233

[B61] Habicher-Schwarz E Pozuzo: Tiroleses, renanos y bávaros en la selva de Perú 2008 Innsbruck: Berenkamp

[B62] INEI Censo 2007 Lima: INEI

[B63] Steinicke E Neuburger M 150 Jahre Tiroler Kolonisten in Peru. Moderner Wandel in Pozuzo am Andenostabhang 2009 Tirol: Tiroler Heimatblätter 50 56

[B64] WHO http://www.who.int/countries/per/en/

[B65] Desmarchelier C Witting Schaus F Sesenta Plantas Medicinales de la Amazonia Peruana. Ecología, Etnomedicina y Bioactividad. Sixty Medicinal Plants from the Peruvian Amazon 2000 Perú: Ecology, Ethnomedicine and Bioactivity

[B66] Desmarchelier C Alonso J Plantas Medicinales para la Atención Primaria de la Salud Vademecum de Fitoterapia 2005 Lima: PRODAPP

[B67] Weller SC Romney AK Systematic data collection 1988 Newbury Park, Calif. [u.a.]: Sage Publ

[B68] Bernard HR Research methods in anthropology qualitative and quantitative approaches 2006 Lanham, Md. [u.a.]: AltaMira Press

[B69] Cotton CM Ethnobotany principles and applications 1997 Chichester [u.a.]: Wiley

[B70] Martin GJ Ethnobotany - a methods manual 2004 London [u.a.]: Earthscan

[B71] Łuczaj Ł Plant identification credibility in ethnobotany: a closer look at Polish ethnographic studies J Ethnobiol Ethnomed 2010 6 1 1 16 10.1186/1746-4269-6-1 21167056 PMC3022638

[B72] Balmé F Plantas Medicinais 1982 Sao Paulo: hemus editora limitada

[B73] Watson Cisneros E Cultivos Tropicales adaprados a la Selva Alta peruana, Particularmente al Alto Huallaga 1985 Lima: Banco Agrario del Peru

[B74] Ferreyra R Flora y Vegetación del Perú Gran Geografia del Peru Naturaleza y Hombre. Volume II 1986 Barcelona: Manfer - Juan Mejía Baca 174

[B75] Balbach A A Flora Nacional na Medicina Natural 1995 1 Edicoes Vida Plena: Plantas Medicinais

[B76] Silva Delgado H García Ruíz A Alvarado Donayre R García Ruíz J Pinedo Panduro M Cerrutti Sifuentes T Plantas Medicinales de la Amazonia Peruana 1995 Iquitos: Instituto Peruano de Seguridad Social, Instituto de Medicina Tradicional

[B77] Mejia K Rengifo E Plantas Medicinales de Uso Popular en la Amazonía Peruana 1995 Lima: Agencia Española de Cooperación Internacional y Instituto de Investigaciones de la Amazonía Peruana (IIAP)

[B78] Castner JL Timme SL Duke JA A Field Guide to Medical and Useful Plants of the Upper Amazon 1998 FL. USA: Feline Press, Gainesville

[B79] Egg Brack A Perú: Diez mil años de domesticación 2003 Lima: Plantas árboles y animales

[B80] Bussmann RW Sharon D Plantas de los cuatro vientos - Las plantas mágicas y medicinales del Perú 2007 Peru: Trujillo

[B81] Fischer MA Exkursionsflora für Österreich, Liechtenstein und Südtirol Bestimmungsbuch für alle in der Republik Österreich, im Fürstentum Liechtenstein und in der Autonomen Provinz Bozen / Südtirol (Italien) wildwachsenden sowie die wichtigsten kultivierten Gefäßpflanzen (Farnpflanzen und Samenpflanzen) mit Angaben über ihre Ökologie und Verbreitung. 3., verb. Aufl. edn 2008 Linz: Land Oberösterreich, OÖ. Landesmuseen

[B82] Egg Schuler A Manual de prácticas agroforestales y silvoculturales en Pozuzo 2009 Peru: IINCAGRO

[B83] Reynel C Pennington RT Pennington TD Flores C Daza A Árboles útiles de la Amazonía Peruana 2003 Peru: Tarea Gráfica Educativa

[B84] Integrated Botanical Information System http://www.anbg.gov.au/ibis/index.html

[B85] Plantas e Ervas Medicinais e Fitoterápicos http://www.plantamed.com.br/

[B86] Tropicos http://www.tropicos.org/

[B87] Borgatti SP ANTHROPAC 4.0 Natick 1996 MA: Analytic Technologies

[B88] Reyes-García V Huanca T Vadez V Leonard W Wilkie D Cultural, practical, and economic value of wild plants: A quantitative study in the Bolivian Amazon Economic Botany 2006 60 1 62 74 10.1663/0013-0001(2006)60[62:CPAEVO]2.0.CO;2

[B89] Borgatti SP Cultural Domain Analysis J Quant Anthropol Kluwer Acad Publishers 1994 4 261 278

[B90] Weller SC Cultural Consensus Theory: Applications and Frequently Asked Questions Field methods 2007 19 4 339 368 10.1177/1525822X07303502

[B91] Vandebroek I The Dual Intracultural and Intercultural Relationship between Medicinal Plant Knowledge and Consensus Economic Botany 2010 1 1520339580

[B92] Borgatti SP ANTHROPAC 4.0 Methods Guide 1996 Natick. MA: Analytic Technologies

[B93] Reyes-Garcia V The relevance of traditional knowledge systems for ethnopharmacological research: theoretical and methodological contributions J Ethnobiol Ethnomed 2010 6 1 32 10.1186/1746-4269-6-32 21083913 PMC2993655

[B94] Signorini M Piredda M Bruschi P Plants and traditional knowledge: An ethnobotanical investigation on Monte Ortobene (Nuoro, Sardinia) J Ethnobiol Ethnomed 2009 5 1 6 10.1186/1746-4269-5-6 19208227 PMC2661884

[B95] Tardío J Pardo-de-Santayana M Cultural Importance Indices: A Comparative Analysis Based on the Useful Wild Plants of Southern Cantabria (Northern Spain) Economic Botany 2008 62 1 24 39 10.1007/s12231-007-9004-5

[B96] Phillips O Gentry A The useful plants of Tambopata. Peru: I. Statistical hypotheses tests with a new quantitative technique Economic Botany 1993 47 1 15 32 10.1007/BF02862203

[B97] Trotter RT Logan MH Etkin NL Informant consensus: A new approach for identifying potentially effective medicinal plants Plants in indigenous medicine and diet 1986 Bedford Hill, New York: Redgrave Publishing Company 91 112

[B98] Heinrich M Ankli A Frei B Weimann C Sticher O Medicinal plants in Mexico: healers' consensus and cultural importance Soc Sci Med 1998 47 11 1859 1871 10.1016/S0277-9536(98)00181-6 9877354

[B99] Collins S Martins X Mitchell A Teshome A Arnason J Quantitative ethnobotany of two east Timorese cultures Economic Botany 2006 60 4 347 361 10.1663/0013-0001(2006)60[347:QEOTET]2.0.CO;2

[B100] Cook FE Royal Botanic G Economic botany data collection standard prepared for the International Working Group on Taxonomic Databases for Plant Sciences (TDWG) 1995 1 Kew: Royal Botanic Gardens

[B101] Voeks R Leony A Forgetting the Forest: Assessing Medicinal Plant Erosion in Eastern Brazil Botany 2004 58 294 306

[B102] de Albuquerque U Soldati G Sieber S Ramos M de Sá J de Souza L The use of plants in the medical system of the Fulni-ô people (NE Brazil): a perspective on age and gender J Ethnopharmacology 2011 133 2 866 873 10.1016/j.jep.2010.11.021 21093569

[B103] Pfeiffer JM Butz RJ Assessing cultural and ecological Variation in ethnobotanical Research: The Importance of Gender J Ethnobiology 2005 25 2 240 278 10.2993/0278-0771(2005)25[240:ACAEVI]2.0.CO;2

[B104] Schunko C Grasser S Vogl C Intracultural variation of knowledge about wild plant uses in the Biosphere Reserve Grosses Walsertal (Austria) J Ethnobiol Ethnomed 2012 8 1 23 10.1186/1746-4269-8-23 22770375 PMC3487943

[B105] Bussmann R Sharon D Traditional medicinal plant use in Northern Peru: tracking two thousand years of healing culture J Ethnobiol Ethnomed 2006 2 1 47 10.1186/1746-4269-2-47 17090303 PMC1637095

[B106] de Jong W Melnyk M Lozano LA Rosales M García M Uña de gato: fate and future of a Peruvian forest resource CIFOR Occasional Paper No. 22 1999 Bogor, Indonesia: CIFOR

